# Complex Three-Dimensional Rearing Environments Amplify Compensatory Plasticity Following Early Blindness

**DOI:** 10.1523/ENEURO.0059-26.2026

**Published:** 2026-07-21

**Authors:** Deepa L. Ramamurthy, Mackenzie Englund, Tanner J. Kovacs, Heather Dodson, Leah A. Krubitzer

**Affiliations:** Center for Neuroscience, University of California, Davis, California 95618

**Keywords:** compensatory plasticity, cross-modal plasticity, environmental enrichment, touch, vibrissa, vision

## Abstract

The neocortex has a remarkable capacity to alter its functional organization and connectivity in response to sensory loss, particularly if this loss occurs early in life. A key question is whether this cross-modal reorganization is driven by sensory deprivation or by enhanced use of the spared senses. We investigated how different rearing environments shape neural responses in primary somatosensory cortex (S1) of short-tailed opossums (*Monodelphis domestica*), following elimination of visual inputs through bilateral enucleation in early development. Early blind and sighted littermates of either sex were reared in enriched environments to promote active tactile exploration in three-dimensional space or in standard laboratory cages. In adulthood, both enriched groups showed adaptive changes in exploration patterns and gap crossing behaviors relative to standard-reared counterparts. Thus, early blind animals showed behavioral compensation when challenged by complex environments. Enriched rearing increased selectivity of S1 neural responses to whisker touch and altered receptive field shapes such that they were less horizontally anisotropic. This shift was strongest in enriched early blind animals, enhancing tuning along the behaviorally relevant horizontal axis more than in standard-reared early blind animals. Thus, alterations in receptive fields of neurons in S1 following early blindness were amplified by environmental complexity. Sighted opossums reared with enrichment also showed similar whisker receptive field plasticity, though to a slightly lower degree. These results demonstrate that the rearing environment strongly influences the reorganization of cortex that processes inputs from the spared senses, underscoring the role of experience in directing compensatory plasticity following early sensory loss.

## Significance Statement

Enhanced perceptual abilities following early sensory loss are often attributed to cross-modal recruitment of cortex linked to the deprived sense. However, plasticity also occurs in cortical areas representing spared modalities. It remains unresolved whether deprivation alone is sufficient to induce such reorganization or whether experience using the spared sense is required. We show that enriched rearing amplifies neural coding changes in primary somatosensory cortex after early blindness, shaping receptive field geometry and promoting adaptive behavioral strategies aligned with environmental demands. Comparable changes in sighted animals reared under the same conditions reveal that reliance on touch—rather than visual deprivation alone—drives this neural and behavioral plasticity, supporting the critical role of experience in enhancing functional outcomes after early sensory impairment.

## Introduction

Early loss of vision leads to heightened perceptual abilities in the remaining senses, accompanied by structural and functional brain plasticity (Goldreich and Kanics, 2003; Voss, 2011; Wong et al., 2011; Lazzouni and Lepore, 2014; Mezzera and López-Bendito, 2016; Morland et al., 2021; López-Bendito et al., 2022). However, the developmental origins of this plasticity remain poorly understood, particularly the extent to which sensory experience, movement options, and affordances contribute to compensatory changes in the brain and behavior. In this study, we investigate how enhancing tactile experience through enriched rearing contributes to neural and behavioral plasticity of whisker-mediated touch in a marsupial, the short-tailed opossum, when vision is lost early in development.

Exposure to enriched environments drives plasticity in sensory cortices of juvenile and adult animals with intact brains and bodies. This includes functional reorganization of sensory representations through changes in cortical territory, neuronal response properties (latency, strength, and selectivity), neuronal morphology, and molecular expression (Leah et al., 1985; Seo, 1992; Coq and Xerri, 1998; Cancedda et al., 2004; Engineer et al., 2004; Polley et al., 2004; Guic et al., 2008; Mégevand et al., 2009; Devonshire et al., 2010; Alwis and Rajan, 2013; LeMessurier et al., 2019; Bibollet-Bahena et al., 2023). Enrichment also counteracts degradation of representations after transient sensory deprivation and promotes recovery from brain injury (Polley et al., 1999; Bartoletti et al., 2004; Sale et al., 2007; Nakashima and Dyck, 2008; Wang et al., 2013; Alwis and Rajan, 2014; Greifzu et al., 2014; Zheng et al., 2014; Zhu et al., 2014; Jiang et al., 2015; Kalogeraki et al., 2017; Levine et al., 2017). Rearing animals in naturalistic environments aligned with a species' lifestyle engages ethologically relevant behavior (Wilkinson et al., 2010; Branchi et al., 2011; Landers et al., 2011; Weissbrod et al., 2013) and is especially effective in reshaping cortical sensory representations at structural, functional, and molecular levels (Polley et al., 1999; 2004; Frostig, 2006; Gomez-Pinilla et al., 2011; Landers et al., 2011). Here, we used a complex three-dimensional (3D) rearing environment designed to model aspects of the natural habitat and lifestyle of short-tailed opossums.

Many small mammals, including short-tailed opossums, routinely move in forest habitats across vertical strata, from floor to canopy (Jenkins, 1974; Jones et al., 2003; Delciellos and Vieira, 2006; Abreu and Oliveira, 2014; Shapiro et al., 2014). Short-tailed opossums are terrestrial hunters and foragers (Jones et al., 2003) but prefer nesting above ground in rocky outcrops (Macrini, 2004), using branches and logs as arboreal runways to negotiate complex terrain (Montgomery, 1980; Ladine and Kissell, 1994; Lammers, 2007; Lammers and Gauntner, 2008). They are skilled climbers despite lacking morphological specializations of arboreal species, relying on behavioral strategies to maintain balance and traverse arboreal substrates (Saunders et al., 1998; Schmitt et al., 2010; Lammers, 2004; 2007; 2009; Lammers and Biknevicius, 2004; Lammers et al., 2006; Lammers and Gauntner, 2008; Wilkinson et al., 2010; Rupert et al., 2014; Goin et al., 2016; Thomas et al., 2017). Whisker touch is critical for navigating spatially complex 3D environments (Ahl, 1986; Arkley et al., 2017), as well as guiding gap crossing and forelimb placement (Arkley et al., 2014; 2017; Niederschuh et al., 2015; Grant et al., 2018). Peripheral organization of the whisker system reflects lifestyle demands and is more developed in arboreal than terrestrial species; in short-tailed opossums, whisker layout is consistent with semiarboreal and scansorial marsupials (Beddard, 1902; Pocock, 1914; Lyne, 1958; 1959; Grant et al., 2013; Muchlinski et al., 2013; Ramamurthy and Krubitzer, 2016; Grant et al., 2017; Grant et al., 2018). Thus, vertical environmental use depends heavily on behavioral modifications guided by tactile input (Lammers and Biknevicius, 2004; Lammers, 2009).

Early loss of visual inputs in short-tailed opossums at Postnatal Day (P4), prior to the formation of retinogeniculate and thalamocortical pathways, enhances tactile acuity and alters neural coding of whisker inputs in primary somatosensory cortex (S1), with receptive fields exhibiting greater selectivity along the principal axis of whisker motion (Ramamurthy and Krubitzer, 2018). Furthermore, there is major reorganization of thalamic and cortical connections of somatosensory cortex in these early blind animals (Dooley and Krubitzer, 2019). This raises a critical question: are alterations in somatosensory coding driven primarily by deprivation-induced anatomical reorganization or by tactile experience itself? Here, we rear animals in conditions that encourage naturalistic tactile exploration in a spatially challenging environment designed to increase reliance on whisker use. By comparing how enrichment shapes neural coding and behavior related to whisker touch in sighted and early blind animals, we interrogate the role of tactile experience in directing compensatory plasticity following early blindness.

## Materials and Methods

### Animals

Thirty-three adult short-tailed opossums of either sex (*Monodelphis domestica*; age range, 4–16 months; weight range, 61–165 g) were used for electrophysiological recording experiments. Animals belonged to four experimental groups (Table 1): standard-reared sighted controls (sSC), standard-reared early blind animals (sEB), enriched-reared sighted controls (eSC), and enriched-reared early blind animals (eEB). Previously published data from standard-reared animals were reanalyzed for comparison with enriched rearing groups (Ramamurthy and Krubitzer, 2018).

**Table 1.** Experimental design

		Visual condition					
		Sighted (SC)			Early blind (EB)		
		Sex	ALL	>2 SD	Sex	ALL	>2 SD
Rearing Condition	Standard (s)	5F, 5M	86	60	4F, 5M	88	64
	Enriched (e)	3F, 4M	84	58	3F, 4M	81	51

Breakdown of subject information for each experimental group. Sexes of animals used for electrophysiological recording experiments, total number of neurons recorded in S1 per group (ALL), and number of S1 neurons with significant whisker-evoked responses (>2 SD) are listed.

### Ethics statement

All protocols were approved by the UC Davis Institutional Animal Care and Use Committee, and experiments were conducted according to the criteria outlined in the National Institutes of Health *Guide for the Care and Use of Laboratory Animals*.

### Bilateral enucleation

**Figure 1.**
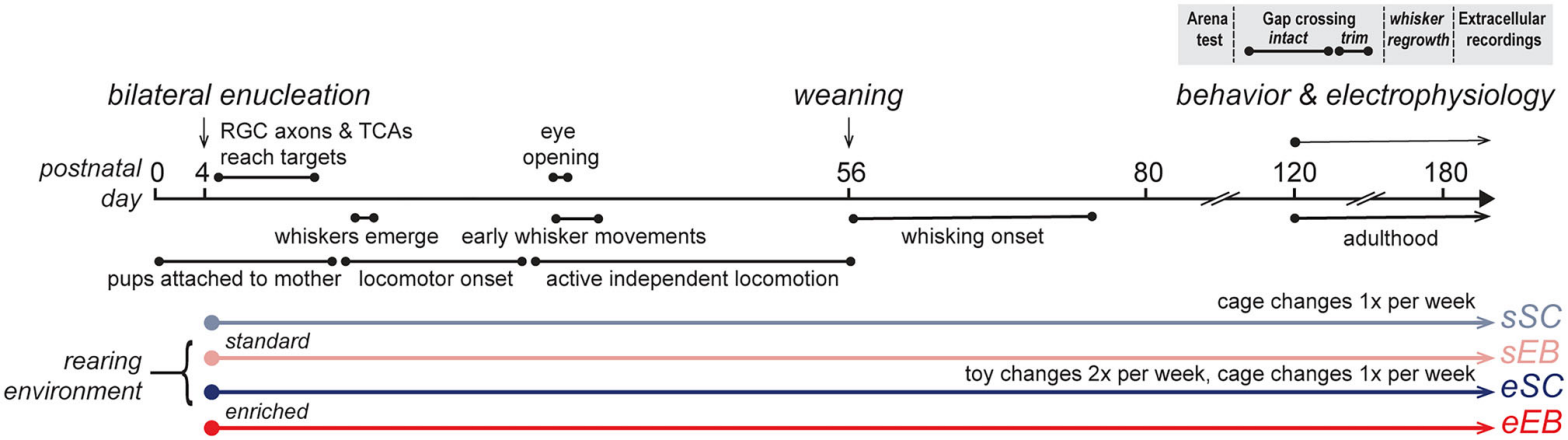
Timeline of experimental manipulations relative to developmental milestones in short-tailed opossums. The four experimental groups are indicated: sSC, sEB (standard-reared early blind), eSC (enriched sighted), and eEB (enriched early blind). Behavioral and electrophysiological experiments were conducted in adults following the sequence shown in the inset, but ages of adult animals were not strictly matched by postnatal day. See also Extended Data Figure 1-1.

For early blind opossums, bilateral enucleations were performed at P4 (Fig. 1). Mothers of experimental litters were lightly anesthetized with Alfaxan (initial dose, 20 mg/kg; maintenance doses, 10–50%, i.m.) to facilitate enucleation of the pups, which are attached to the mother at this developmental stage. Pups were anesthetized by hypothermia. Health of both the mother (respiration rate and body temperature) and the pups (heartbeat, coloration, and mobility) was monitored throughout the procedure. An incision was made in the skin covering the eyes, the eyes were removed under microscopic guidance, and the skin was repositioned and resealed using surgical glue. Approximately 50% of each litter was bilaterally enucleated and the remaining littermates served as sighted controls. After complete recovery from anesthesia, mothers along with their attached litters were placed into either standard or enriched housing, depending on the experimental group.

### Rearing paradigms

Mothers of all experimental groups were reared and housed in standard laboratory cages until bilateral enucleations were performed on the pups at P4. Following the enucleation procedure, mothers with litters belonging to the standard rearing groups were placed in standard laboratory cages, while those belonging to the enriched rearing groups were placed in custom-made enriched cages (Extended Data Fig. 1-1). All animals were maintained on a 14/10 light/dark cycle. Food and water were available *ad libitum* in the cage under both rearing conditions. All experimental litters were housed together with the mother until weaning at ∼8 weeks (P56), and at that stage they were separated from the mother, but the whole litter continued to be housed together until adulthood at ∼4 months (P120). Adults (>P120) were individually housed under standard rearing conditions but in groups of 2–4 same-sex littermates under enriched conditions (see below).

#### Standard rearing

Under standard rearing conditions, adult animals were singly housed in standard laboratory cages, in cages similar to those typically used for laboratory rodents (cage dimensions, 18.5″l × 10″w × 8″h). The home cage was provided with a nesting cup and nesting material (shredded paper towels).

#### Enriched rearing

The enriched rearing environment in our study incorporated features common to environmental enrichment (EE) paradigms previously used in rodents (Nithianantharajah and Hannan, 2006; Sale et al., 2009; Bengoetxea et al., 2012) and additionally takes into consideration the natural lifestyle of short-tailed opossums (Extended Data Fig. 1-1). Juvenile animals were cohoused with the mother and littermates until weaning, and then with just their littermates from weaning until adulthood, as described above. Enriched cages were provided with standard bedding and nesting material, as well as some additional nesting material with a different texture. In addition to the nesting cup placed on the floor as in standard cages, enriched cages provided the option of nesting high above the cage floor, in nesting boxes attached to the cage walls. A single nesting box was provided in the cage at juvenile stages of the experimental litters. In adulthood, balanced numbers of eSC and eEB littermates were housed together in each enriched cage, with a maximum of four opossums per cage. Each adult animal was provided with an individual nesting box. Unlike common enrichment paradigms, which typically incorporate toys into standard cages, the enriched cages used here were substantially larger (∼8× in volume; 29″l × 18″w × 24″h) compared with standard cages to encourage tactile exploration in all three dimensions. The greatest increase relative to standard cages was along the vertical dimension: enriched cages were three times taller than standard cages. Enrichment toys (Bio-Serv) of different shapes and textures were provided in the cage. Toys were replaced, and the positions of manzanita branches were changed every 2–3 d to introduce novelty and promote continual exploration of the environment.

### Behavioral testing

Arena testing: Arena testing was performed in three adult animals from each of the four experimental groups. The arena consisted of a cage with the dimensions of enriched cages, containing a single nest box near the top of the arena, with a branch leading up to it. Animals were placed in the arena and allowed to explore it for 5 min in complete darkness, and movies of their behavior were acquired under infrared (IR) illumination. The idTracker software (Extended Data Fig. 2-1*A*; Romero-Ferrero et al., 2019) was used to extract movement trajectories of individual animals, which was further analyzed in MATLAB. In each movie frame, we segmented the arena into a vertical zone (cage walls, branches, and nest area) and a horizontal zone (cage floor). Exploratory activity was quantified from session-wise binarized matrices (1,280 × 720 pixels; height × width) containing the animal's position in each frame. To assess differences in how animals sampled vertical space in the arena, binarized occupancy maps were summed across sessions separately for standard- and enriched-reared animals and collapsed along the horizontal axis, yielding a per-row estimate of vertical occupancy. Vertical occupancy profiles were then binned in 20-row increments (20 px per bin; vertical dimension only) and normalized to the percentage of total vertical occupancy (summing to 1 across the vertical axis). For each animal, total vertical occupancy (V) and horizontal occupancy (H) were computed by summing binarized pixel values within the corresponding zone across all frames. A normalized vertical preference index was calculated for each animal as follows:

$$\mathrm{Vertical}\;\mathrm{Preference}\;\mathrm{Index}=\frac{\left(V-H\right)}{\left(V+H\right)}.$$

Additional analysis of exploratory behavior in the arena test was performed through manual scoring in BORIS (Behavioral Observation Research Interactive) software. All home cage behavior data were analyzed offline by four independent observers such that multiple epochs were scored by at least two independent observers with a high interrater reliability (Cohen's *κ* coefficient of >0.85; *κ* = 0 indicates no agreement in scoring between observers, and *κ* = 1 indicates perfect agreement; Landis and Koch, 1977; Ramamurthy and Krubitzer, 2018). Arena exploration was initially scored using a binary scale of active states (locomotion bouts) and inactive states (resting bouts). A switch between an active and inactive state was only considered to have occurred if a bout lasted for at least 5 s. Active states were then further scored for crossing events that occurred during natural exploration of the arena. Crossing events were defined as instances in which an animal crossed from one surface to another within the arena. Four types of crossing events were scored: (1) floor to wall/wall to floor, (2) wall to wall, (3) floor to branch/branch to floor, and (4) nest or branch to wall/wall or branch to nest. Since systematic differences were not observed across crossing event types, only combined summary plots are shown.

#### Gap crossing task

Gap crossing was tested in 3–4 adult animals per experimental group. Animals were allowed to spontaneously cross from one platform (“start”) to another platform (“goal”) with gap distances of 2, 4, 6, 8, 10, 12, or 14 cm, which were interleaved in a pseudorandom order across trials. For analysis, these distances were grouped into small (2–10 cm) and large (12–14 cm) conditions. This operational grouping was informed by prior rodent gap crossing literature (Harris et al., 1999; Celikel and Sakmann, 2007; Voigts et al., 2015), together with pilot data in *Monodelphis*, which indicated that animals used their whiskers, rather than other body parts, to make contact with the opposite platform before crossing gaps of 12 cm or greater. Animals were tested for 7 d with intact whiskers, followed by 1–3 d of testing after whisker trimming. On each trial, animals were allowed to remain on the start platform and attempt crossing for a maximum of 1 min. If no crossings were performed, the trial was ended, and animals were removed from the platform for 1 min before returning to the platform for the next trial. Movies of this behavior were acquired under IR illumination or dim red light, and DeepLabCut was used for analysis of gap crossings (Extended Data Fig. 2-1*B*,*C*). The snout tip, the base of the tail, and the position of the edges of each platform were labeled in a subset of frames to generate the dataset (260 frames across 13 opossums) on which the network was trained (200,000 iterations). On a subset of trials, slow-motion movie clips in a zoomed-in field of view were acquired to visually confirm that, during large gap crossings, the goal platform was contacted exclusively by the whiskers prior to crossing and not by the snout or other body parts. Infrared motion sensors (emitter/detector pairs) placed on either side of the edge of each platform detected beam breaks and beam break times were recorded as a secondary method of confirmation.

### Surgical procedures

Animals were anesthetized with urethane (initial dose, 1.25 g/kg, 30% in saline, i.p.; supplemental doses, 0.125–0.313 g/kg, 30% in saline, i.p.), which is well suited for stable long-duration electrophysiological recordings (Silver et al., 2021). Vital signs (respiration and body temperature) were continuously monitored throughout recording sessions to track anesthetic depth and were maintained within comparable ranges across groups (Pagliardini et al., 2012; Silver et al., 2021). At the beginning of the surgery, dexamethasone (0.4–2.0 mg/kg; i.m.) was administered to minimize intracranial swelling. Lidocaine (2% solution, s.c.) was injected at the midline of the scalp and around the ears. The animal was then placed in a stereotaxic frame. Following an incision at the midline of the scalp and retraction of the temporal muscle, a craniotomy was performed to expose the entire parietal cortex and the dura was retracted. The surface of the neocortex was covered with a layer of silicone fluid to prevent desiccation and then photographed so that electrode penetration sites could be related to patterns of vasculature. After the craniotomy, the animal was removed from the stereotaxic frame to permit full access to the face for whisker stimulation. Three stainless steel skull screws were inserted in the skull contralateral to the recording electrode. The head of the animal was stabilized by cementing a head post to the skull screw in a rostral location over the cortex. The other two skull screws were inserted epidurally into the skull over the cerebellum and over the olfactory bulbs, serving as the reference and ground electrodes, respectively, and also providing additional anchoring support for the dental cement.

### Extracellular recordings and whisker stimulation

Somatosensory receptive fields were first coarsely mapped by recording multiunit activity while applying tactile stimuli using a handheld probe. The representation of the whiskers in S1 was localized by progressively testing electrode penetration sites medial to the rhinarium representation and caudal to the lower jaw/lip representation. After this, when receptive fields were found to be located on the mystacial or genal whiskerpad (Ramamurthy and Krubitzer, 2016), computer-controlled whisker deflections were used to quantitatively measure single-unit receptive fields.

Whisker stimuli consisted of single whisker deflections (2°) delivered using piezoelectric actuators (4/100/4 ms ramp-hold-return; interstimulus interval, 1–2 s; 5 mm from the whisker base; 1.4 mm forward excursion). Data were collected for a grid of 16 whiskers on the mystacial pad to enable construction of somatotopic receptive fields. Because whiskers near the edge of the sampled 16-whisker grid have fewer neighboring surround whiskers than more centrally located whiskers, center-versus-edge sampling was closely matched across groups, with each neuron sampled at ∼4 center and 12 edge positions. Center positions were defined as whisker positions for which all eight immediately adjacent surrounding whiskers were also sampled, and all other sampled whiskers were classified as edge positions. Thus, the reported group differences are unlikely to reflect unequal sampling of center-versus-edge whiskerpad positions. Whiskers were deflected in a pseudorandom interleaved order, with 50–100 trials collected per whisker in blocks of 3–50 trials at each recording site.

Extracellular recordings (amplifier gain, 10,000×; A-M Systems Model 1800 Microelectrode AC Amplifier; A-M Systems; sampled at 28 kHz; Power1401, Cambridge Electronic Design) were made 400–500 µm below the pial surface using monopolar tungsten microelectrodes (FHC; 1–5 MΩ at 1 kHz) lowered with a hydraulic microdrive (David Kopf Instruments). This depth corresponds to layer IV based on laminar verification in coronal and tangential sections in which electrolytic lesions together with placement of fluorescent probes marked the electrode locations (Wong and Kaas, 2009; Ramamurthy and Krubitzer, 2018). Recordings were bandpass filtered (300–3,000 Hz), and spike-sorting was carried out offline to confirm isolation of single units. After electrophysiological data were collected, fluorescent probes were used to mark the location of recording sites so that these could be related to histologically identified cortical field boundaries (Extended Data Fig. 4-1).

### Histology

At the end of each experiment, animals were injected with an overdose of sodium pentobarbital (Beuthanasia; 250 mg/kg, i.p.) and transcardially perfused with 0.9% saline, followed by 2–4% paraformaldehyde in phosphate buffer and then 2–4% paraformaldehyde in 10% phosphate-buffered sucrose. The brain was extracted and postfixed (1–2 h in 4% paraformaldehyde in 10% phosphate-buffered sucrose). In some cases, the cortical hemispheres were flattened, and the tissue was sectioned tangentially at 30 μm using a freezing microtome and then processed for myelin staining (Gallyas, 1979; Dooley et al., 2013) to enable reconstruction of cortical field boundaries. Reconstructions were drawn using Adobe Illustrator CS5 (Adobe). Digital images of the processed tissue were obtained using either a Nikon Multiphot system or an Optronics MicroFire digital microscope camera. Contrast and brightness of whole images were adjusted using Adobe Photoshop CS5 (Adobe).

### Neural data analysis

Data files were processed blind to experimental group identity. Single-unit isolation was performed while running the experiment using template-matching procedures in Spike2 (Cambridge Electronic Design; RRID:SCR_000903), and unit isolation was verified offline in Spike2, using principal component analysis. Stable waveforms and firing rates over the course of the recording session and <0.5% refractory period violations (interspike interval <1.5 ms) were required for units to be included in the analysis. Spike times were then exported to MATLAB (MathWorks; RRID:SCR_001622) and analyzed using custom scripts. Whisker-evoked firing rates were measured as the spike count following stimulus onset (0–100 ms) and were considered significant if they exceeded prestimulus spike counts in an equivalent time window by two standard deviations. The best whisker (BW) was defined as the whisker with the highest evoked response, and depending on their proximity to the BW, the remaining whiskers were categorized as first-order surround whiskers (1° SW; immediately adjacent to BW), second-order surround whiskers (2° SW; one position removed from BW), and third-order surround whiskers (3° SW; two positions removed from BW).

One measure of receptive field size used was the number of whiskers that evoked a significant response in each recorded neuron. Another measure used was the rank-ordered whisker tuning curve for each neuron, where responses to all whiskers tested were sorted in a descending order of whisker-evoked response magnitude and normalized to peak (BW response). Furthermore, mean 2D somatotopic receptive fields were obtained after normalizing SW-evoked response magnitudes to the BW-evoked response and aligning receptive fields of single units such that the BW was at the center of the receptive field for each unit. Because *Monodelphis* lacks discrete anatomical cortical barrels (Ramamurthy and Krubitzer, 2016), 2D receptive fields were aligned relative to the BW position rather than an anatomically defined “principal whisker” or “columnar whisker.” The 50, 75, and 90% contour lines (for response levels relative to the BW) were plotted for the mean receptive fields (smoothed by linear interpolation) to facilitate visualization. Raw values were used for all calculations and statistical comparisons.

We define anisotropy in receptive fields as the difference in the tuning width of an S1 neuron's receptive field when measured along different spatial directions of the whiskerpad—along rows (horizontal axis) and along arcs (vertical axis). Anisotropies in the structure of somatotopic receptive fields were quantified as the shape index as follows:

$$\mathrm{Shape}\;\mathrm{index}=\frac{\left(\mathrm{Row}\;\mathrm{tuning}\;\mathrm{width}\;\mathrm{index}-\mathrm{Arc}\;\mathrm{tuning}\;\mathrm{width}\;\mathrm{index}\right)}{\left(\mathrm{Row}\;\mathrm{tuning}\;\mathrm{width}\;\mathrm{index}+\mathrm{Arc}\;\mathrm{tuning}\;\mathrm{width}\;\mathrm{index}\right)}.$$

If the sum of the row and arc tuning width indices was zero, the shape index was assigned to be zero. Shape indices ranged from −1 to 1, with values >0 indicating broader tuning along the row compared with the arc (i.e., broader horizontal tuning) and values <0 indicating broader tuning along the arc compared with the row (i.e., broader vertical tuning). Neuronal selectivity for the best whisker along the row and arc dimensions was measured by assessing tuning width separately along the horizontal and vertical axes as the row tuning width index and the arc tuning width index, respectively, either considering only 1° SW or 1°, 2°, 3° SW in the row or arc of the BW, as stated below:

$$\mathrm{Row}\;\mathrm{tuning}\;\mathrm{width}\;\mathrm{index}=\frac{\left(\mathrm{Mean}\;\;\mathrm{evoked}\;\mathrm{response}\;\mathrm{of}\;SW_{\mathrm{row}}\right)}{\left(\mathrm{BW}\;\mathrm{evoked}\;\mathrm{response}\right)},$$

$$\mathrm{Arc}\;\mathrm{tuning}\;\mathrm{width}\;\mathrm{index}=\frac{\left(\mathrm{Mean}\;\mathrm{evoked}\;\mathrm{response}\;\mathrm{of}\;SW_{\mathrm{arc}}\right)}{\left(\mathrm{BW}\;\mathrm{evoked}\;\mathrm{response}\right)}.$$

Additionally, the whisker selectivity index (WSI) for BW versus in-row or in-arc whiskers was computed separately for each neuron, considering BW responses relative to 1°, 2°, 3° SW responses:

$$\mathrm{WS}I_{\mathrm{row}}=\frac{\left(\mathrm{BW}\;\mathrm{evoked}\;\mathrm{response}-SW_{\mathrm{row}}\;\mathrm{evoked}\;\mathrm{response}\right)}{\left(\mathrm{BW}\;\mathrm{evoked}\;\mathrm{response}+SW_{\mathrm{row}}\;\mathrm{evoked}\;\mathrm{response}\right)},$$

$$\mathrm{WS}I_{\mathrm{arc}}=\frac{\left(\mathrm{BW}\;\mathrm{evoked}\;\mathrm{response}-SW_{\mathrm{arc}}\;\mathrm{evoked}\;\mathrm{response}\right)}{\left(\mathrm{BW}\;\mathrm{evoked}\;\mathrm{response}+SW_{\mathrm{arc}}\;\mathrm{evoked}\;\mathrm{response}\right)}.$$

In-row and in-arc selectivity indices ranged from 0 to 1, with values closer to 1 indicating higher selectivity for the BW in the corresponding dimension.

Receptive field shape was quantified at two spatial scales (1° only vs 1–3°) because somatotopic receptive fields of neurons in the S1 whisker representation consist of the BW (peak response) and a graded, multiwhisker surround shaped by intracortical integration, including anisotropies along row versus arc axes. This receptive field organization has been demonstrated in classical mouse and rat studies (Simons, 1978; Brecht et al., 2003; Fox et al., 2003), as well as prior work demonstrating similar receptive field structure in opossum S1 (Ramamurthy and Krubitzer, 2016; 2018).

Since receptive field shape analysis characterizes the overall geometry of the field around the BW, it cannot be meaningfully computed for a noncontinuous portion of the surround alone (e.g., only 2/3° SWs); therefore 2/3° were not analyzed separately from 1° SWs; instead, shape analyses with only 1° SWs characterized local receptive field configuration, whereas analyses including 1–3° SWs captured receptive field configuration shaped by broader spatial integration.

### Statistical analysis

Statistical analyses were performed in MATLAB and R. Permutation tests for the difference in medians were used to assess differences between two groups (referred to in brief as “permutation test”). Fisher's exact tests (two-sided) were used for analyses of 2 × 2 contingency tables to compare proportions across conditions. All statistical tests for neuronal data use “*n*” of cells. We accounted for interindividual variability using linear mixed-effects models.

To model crossing behavior in the gap-crossing task, we fit a generalized linear mixed-effects model using “fitglme” in MATLAB (Poisson distribution; log link). Crossing counts were modeled with the log of observation time included as an offset to estimate rates. Fixed effects included experimental group, gap size, whisker trimming, and the interactions of experimental group with gap size and with whisker trimming, respectively. Since whisker trimming abolished crossings on 100% of large-gap trials, those trials were excluded from this analysis. Animal identity was included as a random intercept (1|ID) to account for repeated measures. The model specification was as follows:

ResponseVariable∼ExperimentalGroup*Gap+ExperimentalGroup*Trim+(1|AnimalID).

The model was estimated using the Laplace approximation for maximum likelihood. Overdispersion was evaluated via Pearson’s dispersion; if dispersion exceeded 1.5, we added an observation-level random effect (1|Obs) to handle overdispersion.

To analyze gap crossing durations on successful trials, we fit a linear mixed-effects model using “fitlme” in MATLAB with the following specification:

ResponseVariable∼ExperimentalGroup*Gap+ExperimentalGroup*Trim+(1|AnimalID),

where the experimental group and test condition were modeled as fixed effects and animal ID modeled as a random effect.

Planned post hoc comparisons for both models were performed using coefTest with contrast matrices for group and condition effects (gap size and whisker trimming), including a difference-in-differences contrast for the blindness × environment interaction. “coefTest” returned *p* values for each contrast, with Satterthwaite degrees of freedom applied where applicable.

For neural data, the experimental group was modeled as a fixed effect and animal identity as a random effect, using the following model specification:

ResponseVariable∼ExperimentalGroup+(1|AnimalID).

Planned contrasts were performed using “coefTest” for pairwise group comparisons and for a difference-in-differences (blindness × environment) contrast, with *p* values returned and Satterthwaite degrees of freedom applied where applicable. Additional models were fit for key analyses with sex included as a fixed effect to test for differences between males and females, but no significant sex differences were identified.

Exact *p* values for fixed effects of the experimental group and/or test condition (gap size/whisker trimming) obtained from Poisson and Gaussian generalized linear mixed-effects models or from Fisher's exact tests for categorical comparisons with pairwise contrasts across experimental groups are provided in Tables 2 and 3.

**Table 2.** Statistical comparisons for key behavioral results

	Fixed-effect *p* value	Pairwise-comparisons *p* values					
Result/figure panel	Test condition	sSC		sEB		eSC	eEB
% successful trials Figure 3*B* (Holm multiple comparison correction) large versus small intact versus trim (small) intact versus trim (large)
	–	1.26e−139		2.44e−120		6.45e−184	1.04e−155
	–	1		1		1	1
	–	0.017		1.9e−3		3.0e−4	1.4e−3
Number of gap crossings/minute Figure 3*C* large versus small intact versus trim (small)	1.13e−09	1.13e−09		7.82e−10		4.33e−10	9.04e−10
	0.533	–		–		–	–
Gap crossing duration Figure 3*D* large versus small intact versus trim (small)	1.10e−79	8.07e−92		1.47e−08		0.013	0.487
	2.89e−07	0.008		9.61e−09		7.24e−05	0.148
	Expt. Group	sSC versus sEB	sSC versus eSC	sSC versus eEB	sEB versus eSC	sEB versus eEB	eSC versus eEB
% successful trials Figure 3*B* (3) across groups (large, intact)	–	0.675	0.099	0.391	1	1	1
% successful trials Figure 3*B* (3) across groups (small, intact)		1	1	1	1	1	1
% successful trials Figure 3*B* (3) across groups (small, trim)		1	1	1	1	1	1
Number of gap crossings/minute Figure 3*C* (3) across groups (large, intact)	0.556	–	–	–	–	–	–
Number of gap crossings/minute Figure 3*C* (3) across groups (small, intact)		–	–	–	–	–	–
Number of gap crossings/minute Figure 3*C* (3) across groups (small, trim)		–	–	–	–	–	–
Gap crossing duration Figure 3*D* (3) across groups (large, intact)	6.88e−51	7.72e−35	3.65e−62	3.33e−72	3.90e−04	3.20e−09	0.002
Gap crossing duration Figure 3*D* (3) across groups (small, intact)		0.777	0.069	2.91e−14	0.103	1.08e−13	7.55e−05
Gap crossing duration Figure 3*D* (3) across groups (small, trim)		1.33e−05	5.61e−19	6.90e−15	8.62e−06	6.66e−04	0.318

Exact *p* values for fixed effects of experimental group and/or test condition obtained from Poisson and Gaussian generalized linear mixed-effects models or from Fisher's exact tests for categorical comparisons, with pairwise contrasts across experimental groups. See also Extended Data Table 2-1.

10.1523/ENEURO.0059-26.2026.t2-1 Table 2-1 ANOVA marginal tests for fixed effects in Table 2. Download Table 2-1, DOCX file.

**Table 3.** Statistical comparisons for key electrophysiological results

Result/figure panel	Fixed-effect *p* value	Pairwise-comparisons *p* values					
	Expt. Group	sSC vs sEB	sSC vs eSC	sSC vs eEB	sEB vs eSC	sEB vs eEB	eSC vs eEB
Spontaneous activity Figure 4*C*	0.856	–	–	–	–	–	–
Whisker-evoked activity Figure 4*D*	4.54e−10	4.53e−04	4.09e−04	1.11e−11	0.939	1.38e−04	3.04e−04
RF size: number of whiskers Figure 4*E*	1.58e−07	9.43e−07	4.29e−06	8.86e−07	0.832	0.763	0.621
Rank-ordered whisker tuning curve Figure 4*F*	1.55e−25	3.27e−11	1.88e−22	4.04e−19	8.19e−04	0.006	0.618
Shape index: first-order adjacent whiskers Figure 5*C* (left)	0.004	0.249	0.445	6.84e−04	0.628	0.023	0.0033
% horizontal: first-order adjacent whiskers Figure 5*C* Alpha with Bonferroni’s correction = 0.008 (right)	–	0.266	0.185	0.001	0.855	0.039	0.083
Shape index: first- to third-order adjacent whiskers Figure 5*D* (left)	3.45e−05	0.093	0.013	2.19e−06	0.384	0.001	0.016
% horizontal: first- to third-order adjacent whiskers Figure 5*D* Alpha with Bonferroni’s correction = 0.008 (right)	–	0.516	9.88e−05	2.00e−06	7.73e−04	2.50e−05	0.340
% BW response: in-arc whiskers Figure 5*E* (left)	0.042	0.0325	0.0116	0.0244	0.652	0.812	0.848
% BW response: in-row whiskers Figure 5*E* (left)	7.42e−20	7.10e−05	7.59e−14	1.12e−18	6.19e−05	1.25e−08	0.056
BW versus in-arc whiskers Figure 5*F* (left)	0.043	0.080	0.014	0.015	0.436	0.419	0.957
BW versus in-row whiskers Figure 5*F* (right)	2.21e−18	2.86e−04	8.18e−12	7.00e−18	3.03e−04	8.31e−09	0.018

Exact *p* values were obtained from Gaussian generalized linear mixed-effects models or from Fisher's exact tests for categorical comparisons, with pairwise contrasts across experimental groups as indicated (see Materials and Methods). See also Extended Data Tables 3-1 and 3-2.

10.1523/ENEURO.0059-26.2026.t3-1 Table 3-1 ANOVA marginal tests for fixed effects in Table 3. Download Table 3-1, DOCX file.

10.1523/ENEURO.0059-26.2026.t3-2 Table 3-2 ANOVA marginal tests for fixed effects in Figure 5. Download Table 3-2, DOCX file.

## Results

### Behavioral adaptations of blind and sighted opossums in enriched rearing environments

In short-tailed opossums, P0 is developmentally equivalent to embryonic stages in rodents [approximately Embryonic Day (E)11 in mouse and E12 in rat; Molnár and Blakemore, 1995; Molnár et al., 1998]. Bilateral enucleations were conducted at P4, before retinal afferents and thalamocortical axons reached their respective targets (Taylor and Guillery, 1994; Molnár et al., 1998), completely eliminating both spontaneous retinal activity and visually evoked input (Fig. 1). Following bilateral enucleation on P4, opossum mothers and their litters, including sighted and blind pups, were transferred into a new standard or enriched home cage environment (Fig. 1; Extended Data Fig. 1-1*A–D*). Prior to P12, sensory experience in either environment was determined entirely by behavioral patterns of the mother, due to the obligate attachment of opossum pups. Juvenile animals continue to rely on the mother for both sustenance and transportation until weaning at P56, but with increasing independent exploration (Extended Data Fig. 1-1*E*,*F*). Opossum mothers always preferentially nested high above the ground (*n* = 3 mothers, producing a total of 4 enriched experimental litters; example in Movie 1), so pups were forced to rely heavily on touch to navigate the enriched environment. By adulthood, both sighted and early blind animals were highly adapted to their environments (Movies 2, 3).

10.1523/ENEURO.0059-26.2026.f1-1 Figure 1-1 **Standard and enriched rearing environments used for short-tailed opossums. A.** Comparison of cages used in standard (left) and enriched (right) rearing paradigms. Enriched cages (29”l x 18”w x 24”h) were substantially larger (∼8x in volume) than standard cages (8.5”l x 10”w x 8”h), especially in the vertical dimension (∼3x). Each enriched cage contained a nesting box positioned high above the ground, in addition to the standard nesting cup on the cage floor. Cages also included various enrichment objects: regularly repositioned manzanita branches, rotating sets of enrichment toys, a running wheel, and social housing (see *Materials and Methods*). **B.** Frontal view of an enriched cage. **C.** Top view of the same enriched cage shown in **(B).** Letters mark individual components visible in both views: nesting box (a), nesting cup (b), food (c), water (d), running wheel (e), manzanita branches (f–l), and enrichment toys (m). **D.** Mother with an attached experimental litter after placement in the enriched rearing environment. **E–F.** Early blind weanling climbing up a branch to reach the nesting box. Download Figure 1-1, TIF file.

**Movie 1.** A nesting mother opossum navigating the enriched home cage environment. Movie shown at 4× speed. [View online]

**Movie 2.** Sighted opossum in the arena test. Movie shown at 4× speed. [View online]

**Movie 3.** Early blind opossum in the arena test. Movie shown at 4× speed. [View online]

**Figure 2.**
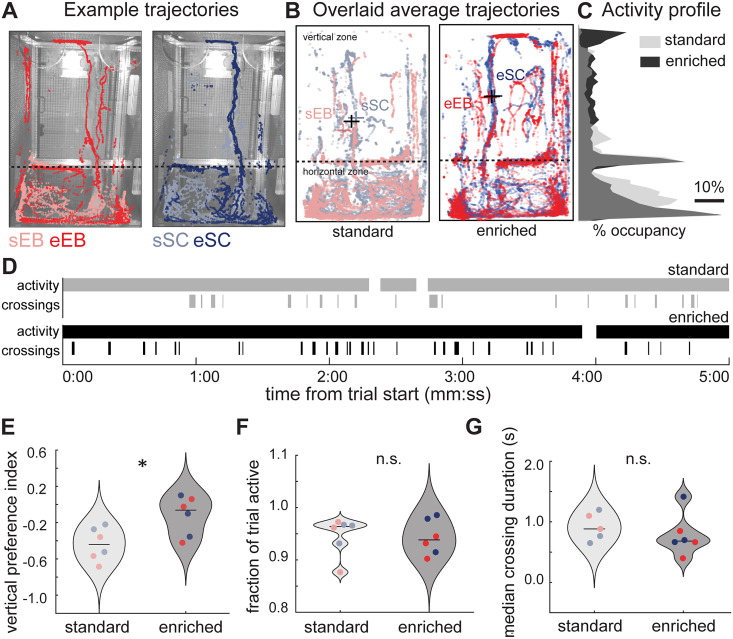
Enriched rearing alters spatial patterns of exploratory behavior. ***A***, Example movement trajectories from one animal in each experimental group (sEB, light red; eEB, dark red; sSC, light blue; eSC, dark blue) tracked over 5 min during behavioral testing in an enriched arena. ***B***, Overlaid movement trajectories from all animals in each experimental group in the arena test show a pattern of increased exploration of vertical versus horizontal space in opossums reared in enriched cages compared with those reared in standard cages. Plus symbols indicate the center of mass of movement trajectories for each group (colors as in ***A***), with data for animals combined within each rearing condition shown in black. ***C***, Vertical occupancy profiles during arena exploration. Normalized distributions of vertical position (height above the arena floor) during exploration derived from 1,280 × 720 px video frames (20 px bins used for visualization), pooled across animals (black, enriched; gray, standard; dark gray denotes regions of overlap between the two distributions). ***D***, Ethograms showing the temporal structure of locomotor activity and spontaneous crossing events during arena exploration. Horizontal bars indicate periods of activity for representative trials from standard-reared (top, gray) and enriched-reared (bottom, black) animals. Vertical tick marks denote spontaneous crossings between surfaces. Time is plotted relative to trial start (mm:ss). ***E***–***G***, Summary behavioral metrics during arena exploration in standard and enriched animals. Distributions of vertical preference index (***E***), fraction of trial time spent active (***F***), and median crossing duration (***G***). Violin plots show Gaussian kernel density estimates of the pooled distribution for each category; horizontal lines denote medians. Points represent individual animals, color-coded by the experimental group. Animals reared in the enriched environment show a significant increase in vertical preference index. Overall activity levels and duration of crossing events during free exploration were not significantly different. See also Extended Data Figure 2-1.

To assess how rearing condition and visual condition (blind vs sighted) shape exploratory behavior, we tested each group in a novel arena matching the dimensions and basic layout of the enriched home cages (Fig. 2; Extended Data Fig. 2-1*A*). We quantified spatial sampling preferences (Fig. 2*A*–*C*,*E*), overall locomotor activity (Fig. 2*D*,*F*), and spontaneous crossings between surfaces during natural exploration (Fig. 2*D*,*G*), capturing both spatial and temporal aspects of behavior. These measures contextualize and complement the more controlled gap crossing behavior assay presented in the next section. Movement trajectories were highly similar for sighted and blind animals within each rearing condition (Fig. 2*A*,*B*; Extended Data Fig. 2-1*A*), consistent with successful behavioral compensation by blind animals even in more spatially complex environments. In contrast, rearing environment strongly impacted how animals sampled the novel arena: enriched opossums explored vertical space more extensively than those reared in standard cages (*p* = 0.018, permutation test; Fig. 2*C*,*E*), though overall activity levels (*p* = 0.565, permutation test) and durations of naturally occurring crossing events were not significantly different (*p* = 0.232, permutation test; Fig. 2*D*,*F*,*G*). Quantifying gap crossing during free exploration has some limitations which may mask differences in sensory-mediated behavior. Crossing events that occurred during free exploration, where animals crossed from surface to surface did not necessarily involve crossing of discrete gaps, and we could not control for the gap size across different crossing events. Further animals were free to adopt varying postures prior to crossing and to use varying behavioral strategies involving sensory inputs from multiple body parts to aid gap crossing during free exploration.

10.1523/ENEURO.0059-26.2026.f2-1 Figure 2-1 **Behavioral movie analyses. A.** Representative frame showing semi-automated identification of an opossum during arena testing using idtracker.ai. **B.** Representative frame showing behavioral tracking in the gap crossing task using DeepLabCut. “IR1” and “IR2” indicate the positions of infrared motion sensors mounted on the edge of each platform, tracked with DeepLabCut to aid in gap crossing analyses. **C.** Zoomed-in view showing an opossum on the start platform extending its whiskers toward the goal platform prior to crossing the gap. Download Figure 2-1, TIF file.

**Figure 3.**
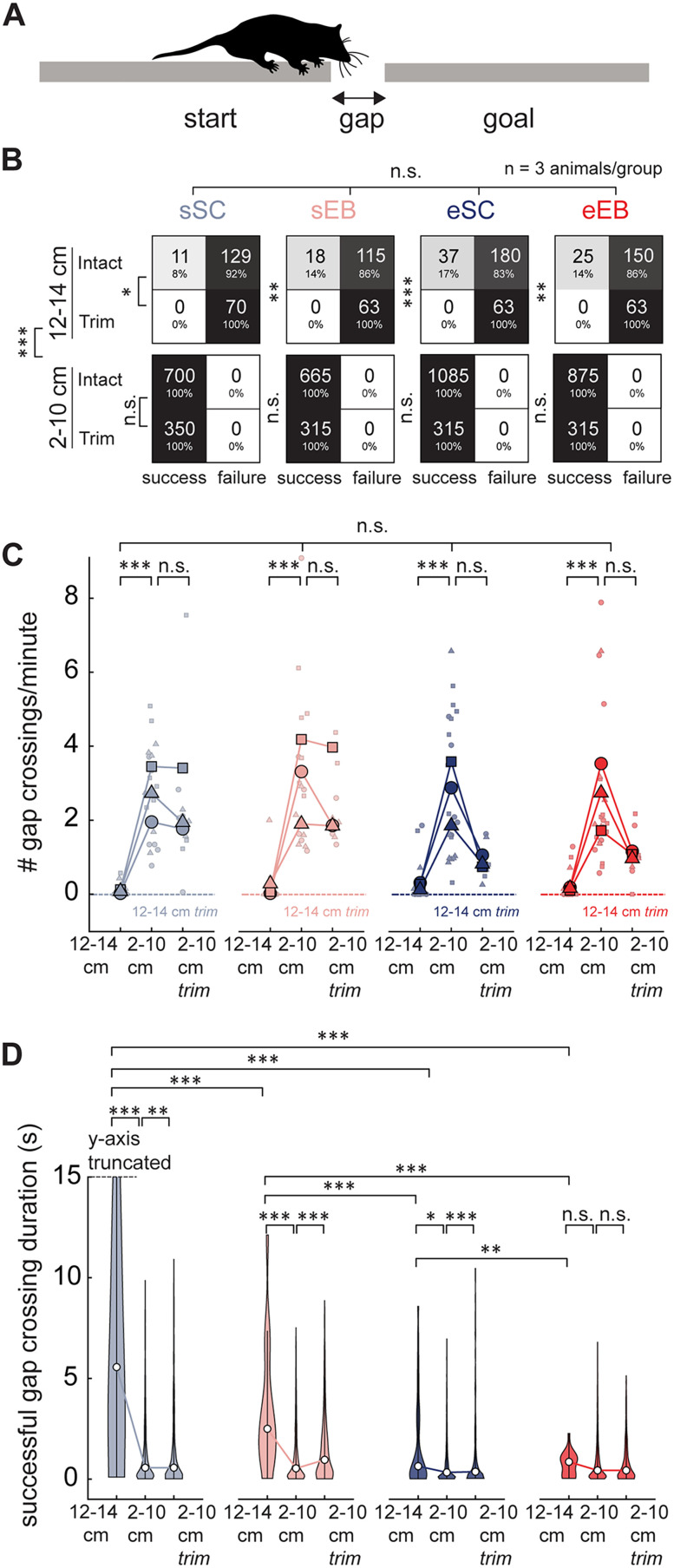
Enriched rearing alters sensorimotor performance during gap crossing. ***A***, Experimental setup in the gap crossing task. ***B***, Contingency table summaries of gap crossing outcomes for small (2–10 cm) and large (12–14 cm) gaps under intact and whisker-trimmed conditions, shown separately for each experimental group (sSC, sEB, eSC, eEB). Columns indicate success and failure, rows indicate intact and trimmed conditions, and each cell shows the raw number of trials with the corresponding percentage indicated in grayscale (0%, white; 100%, black). Numbers in each box indicate the percent of successful trials. ***C***, Gap crossing rates (crossings/minute) for large gaps (12–14 cm), small gaps (2–10 cm), and small gaps after whisker trimming. Large markers (circle, triangle, square) show individual animals, connected by lines across conditions; smaller faded markers of matching shape show the underlying session-level values. Three animals are shown per group, corresponding to those represented in all plotted conditions. Data for large gaps after whisker trimming are not shown, as no successful crossings occurred in this condition. ***D***, Duration of successful crossings for large and small gaps (with intact or trimmed whiskers). Most groups show significantly longer durations for successful gap crossing on large gaps (12–14 cm) versus small gaps (2–10 cm). Upon whisker trimming, most groups show a significant increase in gap crossing duration for small gaps, consistent with the use of whisker-dependent strategies for gap crossing. Enriched blind animals were unique in showing no significant difference between large and small gap durations and no trimming-related increase in small gap crossing times, reflecting enhanced performance on large gaps through whisker use alongside compensatory tactile strategies using other body parts that preserve performance on small gaps when whiskers are absent. For larger gaps, there are prominent differences between experimental groups based on rearing condition and blindness. Early blind animals crossed gaps faster than sighted controls in standard conditions, and their performance was further enhanced by enriched rearing. Violin plots (color-coded by the experimental group) show Gaussian kernel density estimates of the data distribution, with medians indicated by circular markers. See also Extended Data Figure 2-1, Table 2, and Extended Data Table 2-1.

To assess sensorimotor performance under more precisely defined task parameters, we used a gap crossing task (conducted in darkness) in which the gap size was systematically varied across trials, animals had only one possible path forward (across the gap), and the constraints of the apparatus required animals to adopt a standardized posture prior to gap crossing (Fig. 3*A*; Fig. 2-1*B*,*C*). Crossing large gaps placed animals in a whisker-dependent regime, as the goal platform was out of reach from the start platform except through whisker contact. We quantified (1) the fraction of trials resulting in a successful crossing (Fig. 3*B*), (2) crossing rates over time (Fig. 3*C*), and (3) the duration of successful crossing events (Fig. 3*D*).

The fraction of successful trials (i.e., the probability of success) was strongly impacted by gap size—with intact whiskers, animals crossed successfully on only 8–17% of large gap trials across groups (sSC, 8%; sEB, 14%; eSC, 17%; eEB, 14%; no significant differences across groups), compared with 100% of small gap trials (Fig. 3*B*, Fisher's exact tests, *p* < 0.001 for all groups; no significant differences across groups; see Table 2 for exact *p* values). Furthermore, whisker trimming completely eliminated successful crossings at large gaps in all experimental groups. This is consistent with the role of whiskers in gap crossing described in previous studies of small mammals (Hutson and Masterton, 1986; Voigts et al., 2015; Arkley et al., 2017; Graham and Socha, 2020; Diamond and Toso, 2023). All animals crossed on 100% of small gap trials, with or without whiskers, indicating that other body parts also contributed to small gap crossings.

Crossing rates, which reflect success rate normalized by time, were significantly lower for large gaps than for small gaps within each experimental group (Fig. 3*C*; Table 2; Extended Data Table 2-1; *p* < 0.001 in all cases, all *p* values from Poisson generalized linear mixed-effects model) with no significant differences across groups (*p* > 0.05). Although there was a trend toward decreased crossing rates in all experimental groups with whisker trimming, trimming effects were not statistically significant for crossing rates (*p* > 0.05).

The third metric in the gap crossing task was the duration of successful crossings (Fig. 3*D*; Table 2; Extended Data Table 2-1). We observed significant fixed effects of the experimental group (*p* < 0.001; all *p* values from the linear mixed-effects model), gap size (*p* < 0.001), and whisker trimming (*p* < 0.001), as well as interactions between group and gap size (*p* < 0.001) and between group and trimming (*p* < 0.05). Across groups, successful crossings were generally slower for large versus small gaps, with modest but significant increases in crossing times on small gaps after whisker trimming (with one exception; see below). Consistent with Figure 3*B*, this pattern suggests primary reliance on whiskers for large gaps, whereas small gaps can be crossed using strategies that incorporate both whiskers and tactile inputs from other body parts (Ramamurthy et al., 2021).

Group differences based on visual condition and rearing condition were most pronounced for large gaps (Fig. 3*D*). Early blind opossums crossed large gaps faster than sighted controls under standard rearing (sSC vs sEB, *p* < 0.001), and this advantage was amplified by enriched rearing (eEB vs sEB, *p* < 0.001). Both enriched groups were significantly faster than standard-reared animals (*p* < 0.001 for all pairwise comparisons), with enriched blind animals outperforming enriched sighted animals (*p* < 0.01). Enriched blind animals followed the general trends of other groups but were the only cohort without a significant difference between large and small gap durations (*p* > 0.05), reflecting comparatively faster large gap performance. They were also the only group in which whisker trimming did not significantly prolong small gap crossings (*p* > 0.05). Taken together, these results indicate heightened performance supported by whisker use on large gaps, along with adaptive recruitment of other body parts to maintain small gap performance when whiskers are absent.

### Tactile experience amplifies compensatory neural plasticity in blind opossums

**Figure 4.**
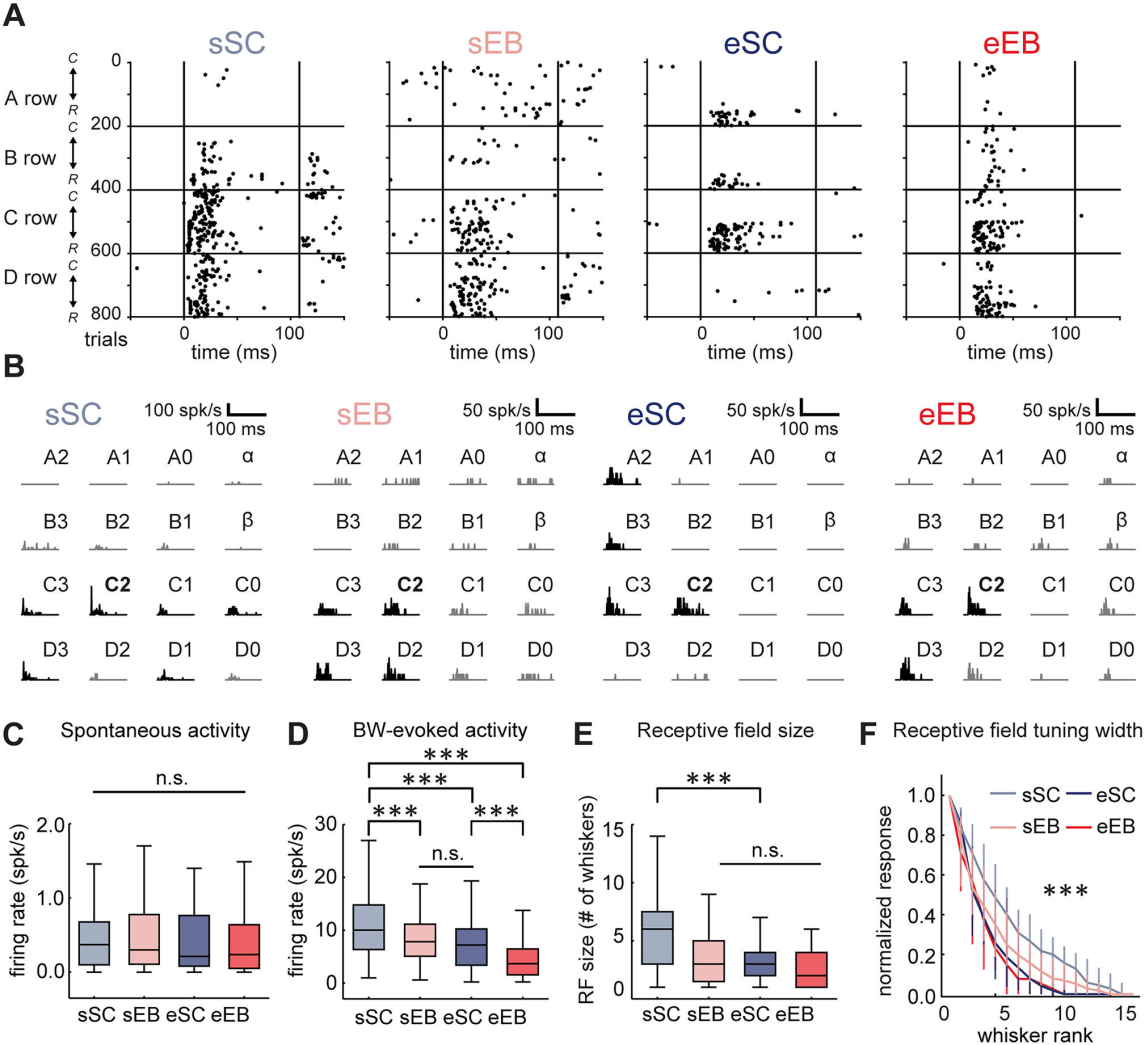
Enriched rearing amplifies alterations in S1 neuron responses to whisker touch in early blind animals and produces similar shifts in sighted controls. ***A***, Spike raster plots from example neurons responsive to whisker touch in each of the four experimental groups (left to right, sSC, sEB, eSC, eEB). Examples are shown for neurons with the same best whisker (BW), C2. Trials corresponding to ***A***, ***B***, ***C***, and ***D*** whisker rows are separated by horizontal lines. Within each row, the direction of more rostral (“R”) versus more caudal (“C”) whiskers is indicated on the *y*-axis. ***B***, PSTHs for the same neurons shown in ***A***. PSTHs corresponding to whiskers with response magnitudes >50% of the BW response are plotted in black; all others are in gray. The examples in ***A*** and ***B*** illustrate more selective responses to whisker touch in standard-reared blind opossums, which is further amplified in enriched groups. Additionally, neural responses to whiskers vertically adjacent to the BW are stronger than those for horizontally adjacent whiskers in enriched groups, especially in enriched early blind opossums. ***C***, Spontaneous firing rates of S1 neurons were not significantly different across experimental groups. ***D***, Firing rates evoked by the BW showed significant differences across experimental groups. Notably, both sighted and early blind enriched groups showed suppression relative to sSC, similar to effects previously reported in early blind animals under standard rearing conditions. The magnitude of suppression of the BW-evoked response was greatest in early blind animals reared in the enriched environment. ***E, F***, Mean receptive field size was significantly smaller in sEB, eSC, and eEB animals relative to sSC. This is seen both in terms of number of whiskers driving significant responses (***E***) and the rank-ordered tuning curve (***F***) for whisker-responsive S1 neurons. See also Table 3, Extended Data Table 3-1, and Extended Data Figure 4-1.

Single-unit recording experiments were performed in 19 opossums reared in standard conditions (10 sSC, 9 sEB) and 14 opossums reared in enriched conditions (7 eSC, 7 eEB), acquiring data for a total of 339 neurons across experimental groups (Table 1; Extended Data Fig. 4-1). Physiological measures monitored during electrophysiological recordings were not significantly different across groups (mean respiration rate, breaths/min: sSC, 48.6 ± 3.98; sEB, 50.4 ± 3.59; eSC, 49.7 ± 3.70; eEB, 48.7 ± 2.52; mean body temperature, °F, sSC, 91.2 ± 0.158; sEB, 91.1 ± 0.167; eSC, 91.1 ± 0.201; eEB, 91.2 ± 0.151). The 63–73% of units recorded in S1 (sSC, 60; sEB, 64; eSC, 58; eEB, 51) had a significant evoked response (>2 SD above baseline) to stimulus onset following single whisker deflections, and these neurons were included in receptive field analyses. Raster plots and peristimulus time histograms (PSTHs) for whisker-responsive neurons in each group are shown in Figure 4, *A* and *B*. These examples illustrate more selective whisker touch responses in standard-reared blind opossums, with selectivity further amplified in enriched groups.

10.1523/ENEURO.0059-26.2026.f4-1 Figure 4-1 **Histological verification of recording sites. A, C, E, G.** Myelin-stained tangential sections of cortex from a standard-reared sighted (sSC) animal **(A)**, a standard-reared early blind (sEB) animal **(C)**, an enriched sighted (eSC) animal **(E)**, and an enriched early blind (eEB) animal **(G)**. Primary somatosensory cortex (S1) is identifiable as a darkly staining region relative to adjacent cortical fields. **B, D, F, H.** Reconstructions of myeloarchitectural borders drawn from the series of myelin-stained sections in sSC (**B**), sEB (**D**), eSC (**F**) and eEB (**H**) animals. The locations of fluorescent probe insertions are shown as open circles, and recording sites are shown as black filled circles. Only sites at which whisker-evoked neuronal responses were quantified are included. In all panels, medial (M) and rostral (R) directions are indicated by arrows. Scale bar = 250 µm. Download Figure 4-1, TIF file.

In general, spontaneous firing rates of S1 neurons were low, consistent with previously reported findings in standard-reared animals (Ramamurthy and Krubitzer, 2018) and were not significantly different among experimental groups (Fig. 4*C*; Table 3; Extended Data Table 3-1; mean ± SEM; sSC, 0.582 ± 0.083 spk/s; sEB, 0.613 ± 0.121 spk/s; eSC, 0.544 ± 0.079 spk/s; eEB, 0.503 ± 0.082 spk/s; linear mixed-effects model, *p* > 0.05; see Table 3 for exact *p* values for the fixed-effect of the experimental group and for all pairwise comparisons between groups). However, responses evoked by the stimulation of even the most preferred single whisker stimulus (best whisker; BW) in enriched groups were suppressed in relation to stimulus-evoked firing rates in standard-reared sighted opossums (Fig. 4*D*; Table 3; Extended Data Table 3-1; mean ± SEM; sSC, 11.4 ± 0.839 spk/s; sEB, 8.39 ± 0.591 spk/s; eSC, 7.82 ± 0.748 spk/s; eEB, 4.57 ± 0.52 spk/s; linear mixed-effects model; eSC vs sSC, *p* < 0.01; eEB vs sSC, *p* < 0.001), as previously reported in early blind animals under standard rearing conditions (Ramamurthy and Krubitzer, 2018). Since relative suppression of BW-evoked responses was observed in enriched sighted controls, in addition to enriched early blind animals, this effect was not contingent on the lack of vision itself—rather it was driven by increased dependence on touch in the enriched groups of opossums. Mean BW-evoked firing rates in enriched sighted control animals were comparable to those in standard early blind animals (linear mixed-effects model, sEB vs eSC, *p* > 0.05). However, in enriched early blind animals, mean BW-evoked firing rates were lower relative to all other experimental groups (linear mixed-effects model, eEB vs sEB, *p* < 0.001; eEB vs eSC, *p* < 0.001; eEB vs sSC, *p* < 0.001). Thus, blindness was not required for suppression of whisker-evoked responses, but the combination of tactile enrichment and vision loss produced the greatest effect on whisker-evoked firing rates of S1 neurons. This is consistent with animals relying on their whiskers to a greater extent when reared in a complex environment in the absence of vision.

Average whisker receptive fields in both sighted and blind enriched groups were significantly smaller than those in sSC (mean ± SEM, eEB, 3.22 ± 0.409; eSC, 3.48 ± 0.364; sSC, 5.92 ± 0.427; linear mixed-effects model, eSC vs sSC, *p* < 0.001; eEB vs sSC, *p* < 0.001) when measured as the number of whiskers that evoked a significant response above spontaneous firing (Fig. 4*E*; Table 3; Extended Data Table 3-1). Because enriched groups also showed reduced whisker-evoked response magnitudes, the smaller receptive fields measured by the number of significantly responsive whiskers may in part result from less of the receptive field exceeding response threshold. To address this possibility, we also examined receptive field measures based on responses to all whiskers tested and normalized to peak response. We see similar results when receptive fields are quantified as the mean rank-ordered tuning curve across neurons (Fig. 4*F*; Table 3; Extended Data Table 3-1; linear mixed-effects model, eSC vs sSC, *p* < 0.001). The finding that tactile enrichment in sighted control animals leads to a decrease in mean receptive field size—similar to both standard-reared and enriched early blind animals—supports the interpretation that this reduction reflects an experience-dependent effect across all three groups. However, changes in measures of overall receptive field size in enriched early blind animals relative to sEB were either not significant (Fig. 4*E*, number of whiskers, eEB, 3.22 ± 0.409; sEB, 3.38 ± 0.270; linear mixed-effects model, eEB vs sEB, *p* > 0.05) or modest (Fig. 4*F*, rank-ordered tuning curve, linear mixed-effects model, *p* < 0.01). This suggests that, under standard rearing conditions, receptive field plasticity in early blind animals may already have reached a limit that cannot be further shifted by enrichment.

### Enrichment enhances selectivity for whisker touch along the behaviorally relevant horizontal axis

**Figure 5.**
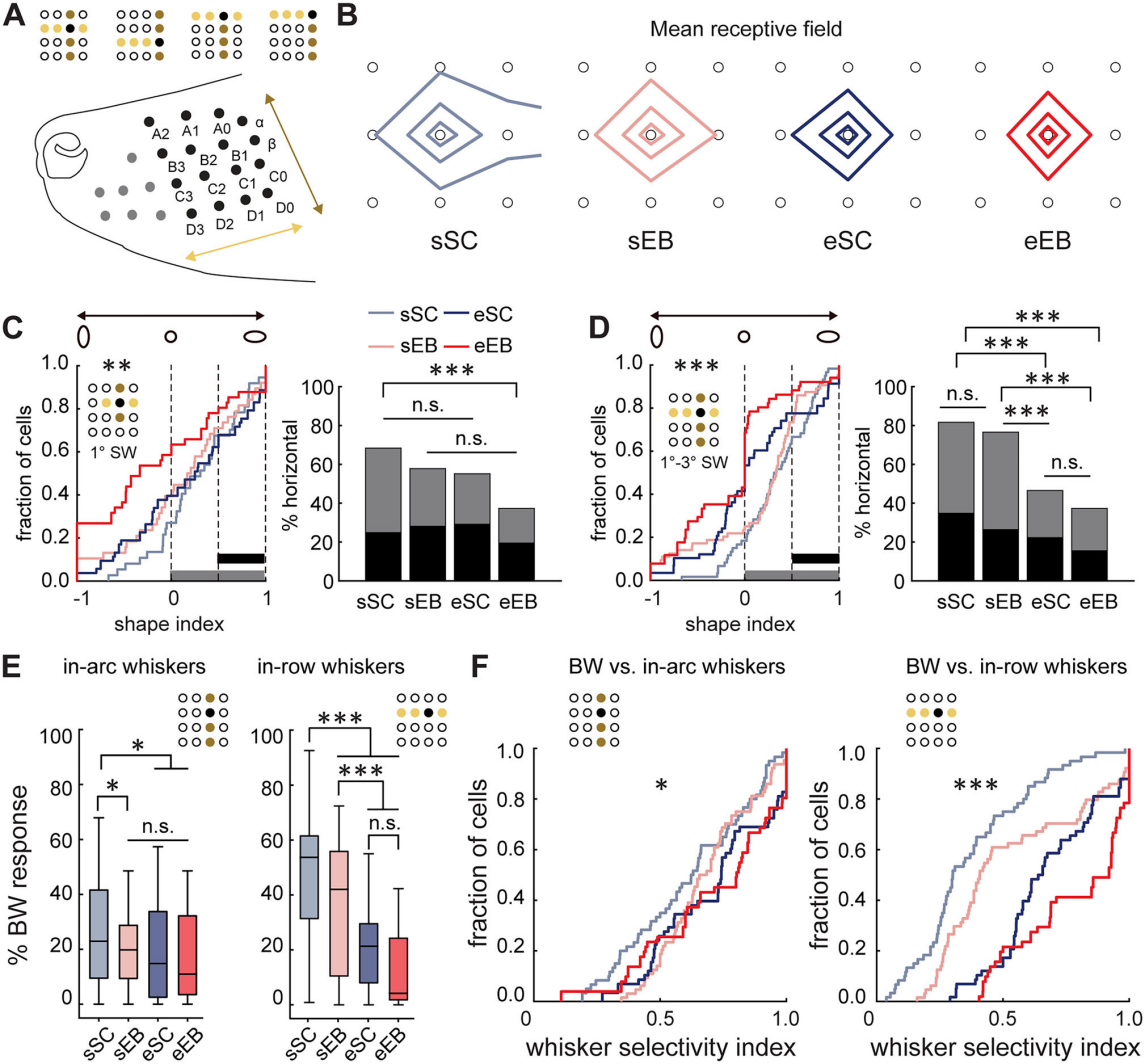
Enriched rearing amplifies anisotropies in receptive fields of S1 neurons in early blind animals, improving selectivity for whisker touch along a behaviorally relevant axis. ***A*,** Schematic of the opossum mystacial whiskerpad indicating whisker rows (light yellow) and arcs (gold) and the horizontal and vertical axes used for receptive field shape analyses. Insets in individual panels depict the grid of 16 whiskers and the SW positions included in each analysis, shown for one example best whisker (BW) position (black filled circle). ***B***, Mean 2D somatotopic receptive fields for each experimental group (left to right, sSC, sEB, eSC, eEB), shown with 50, 75, and 90% contour lines (response levels relative to the BW, smoothed by linear interpolation for display). This visualization highlights the degree of horizontal versus vertical spread (anisotropy) in the average receptive field configuration for each experimental group. ***C, D*,** Receptive field shape distributions for S1 neurons in enriched animals were shifted away from horizontal anisotropic shapes, which are typical of standard-reared animals. ***C***, Receptive field shape distributions when only 1° SW positions were considered (left) and fractions of horizontally anisotropic receptive fields corresponding to each group (right). Light gray bars indicate horizontally elongated receptive fields defined as shape indices >0, while the black portions within each bar indicate horizontally elongated receptive fields with a stricter cutoff of shape indices >0.5. ***D***, Data for the same neurons when all SW positions (1–3°) were included. Horizontally anisotropic receptive fields are decreased in both enriched groups of animals and to the greatest extent in enriched early blind animals. ***E***, Quantification of tuning width along the row (horizontal/rostrocaudal axis) and arc (vertical axis) across experimental groups. Enriched groups show sharper tuning along the horizontal axis, to a significantly greater extent than sEB. Enriched groups also show sharper tuning along the vertical axis compared with sSC, but only to the same extent as previously reported in sEB. ***F***, Distributions of the whisker selectivity index of S1 neurons calculated separately for in-arc whiskers (left) and in-row whiskers (right), when including all SW positions (1–3°). sEB, eSC, and eEB groups all showed significantly greater selectivity for whiskers within arcs, but were not significantly different from each other. The greatest increases in whisker response selectivity were seen for whiskers within rows, i.e., the horizontal/rostrocaudal axis, which is the primary axis of natural whisker motion. Selectivity was greatest for eEB animals followed by eSC and sEB animals, with all three of these groups showing significantly higher selectivity along the horizontal axis compared with sSC animals. See also Table 3 and Extended Data Table 3-2.

To better understand the effects of enriched rearing on receptive field configuration of S1 neurons, we characterized 2D receptive fields for the row (horizontal) and arc (vertical) dimensions of the whiskerpad (Fig. 5*A*). Visual inspection of mean 2D receptive fields revealed clear differences in receptive field anisotropies across experimental groups (Fig. 5*B*). We quantified anisotropies in receptive fields as the shape index, ranging in value from −1 to 1, where positive values indicated horizontally anisotropic shapes, negative values indicated vertically anisotropic shapes, and values close to 0 indicated more isotropic shapes. Receptive fields for neurons in the S1 whisker representation in sSC and early blind animals were dominated by horizontally anisotropic shapes (Fig. 5*C*,*D*; Table 3; Extended Data Table 3-2). However, for early blind animals reared in enriched conditions, the receptive field shape distributions were shifted away from horizontal anisotropy—either when considering only 1° SW positions (Fig. 5*C*; neurons with shape index >0.0; sSC, 68.3%; sEB, 57.8%; eEB, 37.3%; Fisher's exact test, eEB vs sSC, *p* = 0.001; eEB vs sEB, *p* < 0.05; linear mixed-effects model, eEB vs sSC, *p* < 0.001; eEB vs sEB, *p* < 0.05) or when including all SW (1–3°) positions (Fig. 5*D*; neurons with shape index >0.0; eEB, 37.3%; sSC, 81.7%; sEB, 76.6%; Fisher's exact test, eEB vs sSC, *p* < 0.001; eEB vs sEB, *p* < 0.001; linear mixed-effects model, eEB vs sSC, *p* < 0.001; eEB vs sEB, *p* = 0.001).

Receptive field distributions were also shifted away from horizontal anisotropy in sighted control animals reared in the enriched environment. This shift was not seen when only 1° SW positions were considered (Fig. 5*C*; neurons with shape index >0.0; eSC, 55.2%; Fisher's exact test, eSC vs sSC, *p* > 0.05; eSC vs sEB, *p* > 0.05; linear mixed-effects model, eSC vs sSC, *p* > 0.05; eSC vs sEB, *p* > 0.05) but becomes apparent when 1–3° SW positions are considered (Fig. 5*D*; neurons with shape index >0.0, eSC, 46.6%; Fisher's exact test, eSC vs sSC, *p* < 0.001; eSC vs sEB, *p* < 0.001; linear mixed-effects model, eSC vs sSC, *p* < 0.05; eSC vs sEB, *p* > 0.05). Thus, rearing in an enriched environment, which was permissive of more naturalistic patterns of tactile behavior, shifted the shapes of receptive fields of S1 neurons away from the horizontally anisotropic shapes typically found in animals reared in standard laboratory cages. The shift occurred in both sighted and early blind enriched animals, but the effect was more pronounced in early blind animals.

The results of receptive field shape analyses pointed to differential effects of rearing environment on tuning along horizontal and vertical axes of receptive fields. We quantified neuronal tuning width separately along whisker rows and arcs for each experimental group (Fig. 5*E*; Table 3; Extended Data Table 3-2). Both sighted and early blind enriched animals showed sharper tuning along both the vertical and horizontal axes relative to sighted controls, but there was a striking difference in the magnitude of tuning width changes along the horizontal versus vertical axes.

This constitutes an amplification of shifts in receptive field anisotropies previously described in early blind animals under standard rearing conditions (Ramamurthy and Krubitzer, 2018). Along the arc (vertical) axis, tuning was sharpened to the same extent in the enriched groups as seen in sEB relative to sighted controls (Fig. 5*E*; %BW response for in-arc whiskers, eEB, 19.4 ± 2.80%; eSC, 18.7 ± 2.13%; sEB, 20.1 ± 1.58%; sSC, 26.7 ± 2.50%; linear mixed-effects model, eEB vs sSC, *p* < 0.05; eEB vs sEB, *p* > 0.05; eSC vs sSC, *p* < 0.05; eSC vs sEB, *p* > 0.05). However, along the row (horizontal axis), both sighted and early blind enriched animals showed sharper tuning relative to both groups of standard-reared animals (Fig. 5*E*; %BW response for in-row whiskers, eEB, 13.8 ± 2.07%; eSC, 20.9 ± 1.96%; sEB, 35.1 ± 2.82%; sSC, 49.1 ± 2.85%; linear mixed-effects model, eEB vs sSC, *p* < 0.001; eEB vs sEB, *p* < 0.001; eSC vs sSC, *p* < 0.001; eSC vs sEB, *p* < 0.001). There was a trend for sharper row tuning in enriched early blind animals compared with enriched sighted control animals, but this was not significant (linear mixed-effects model; eSC vs eEB, *p* > 0.05). We computed the whisker selectivity index as the normalized difference ratio of the neuronal response to the best whisker in comparison with responses to in-row or in-arc whiskers. Population distributions of the selectivity index for S1 neurons (Fig. 5*F*; Table 3; Extended Data Table 3-2) further validate the anisotropy in the enhancement of selectivity seen for animals reared in enriched environments (BW vs in-arc whiskers, linear mixed-effects model, eEB vs sSC, *p* < 0.05; eEB vs sEB, *p* > 0.05; eSC vs sSC, *p* < 0.05; eSC vs sEB, *p* > 0.05; eEB vs eSC, *p* > 0.05; BW vs in-row whiskers, linear mixed-effects model, eEB vs sSC, *p* < 0.001; eEB vs sEB, *p* < 0.001; eSC vs sSC, *p* < 0.001; eSC vs sEB, *p* < 0.001; eEB vs eSC, *p* < 0.05).

Thus, a spatially complex enriched environment drives significantly greater improvement in neuronal selectivity for whisker touch in early blind animals especially along the rostrocaudal axis, which is the primary axis of natural whisking behavior. A similar improvement in selectivity was observed in sighted control littermates reared in the enriched environment, supporting the role of tactile experience rather than vision loss itself in driving these effects. In summary, the results of our current study indicate that compensatory effects on neural coding in S1 following early blindness do not arise solely from visual deprivation and can be amplified and directed by tactile experience.

## Discussion

Although the effects of enrichment on plasticity within a sensory modality have been studied at structural, functional, and molecular levels, few studies have examined its impact on cross-modal compensatory plasticity, focusing mainly on c-fos expression or synaptic changes (van Praag et al., 2000; Piche et al., 2004; Mundiñano and Martínez-Millán, 2010; Zheng et al., 2014; Lee, 2023). The short-tailed opossum is born with a highly immature nervous system, enabling precise extrauterine manipulations at very early stages in development (Ramamurthy and Krubitzer, 2018; Ramamurthy et al., 2021). Here, we tested the effects of rearing sighted and early blind short-tailed opossums in a large and dynamic, spatially complex 3D environment and examined effects on tactile behavior and receptive field configurations of neurons in S1.

Enrichment reduces abnormal stereotypic behaviors (e.g., overgrooming) and other behavioral indicators of stress and aggression and enhances learning, memory, motor performance, and social behavior (Mason et al., 2007; Amaral et al., 2008; Girbovan and Plamondon, 2013; Bailoo et al., 2018; Khoo et al., 2020; Kentner et al., 2021; Mieske et al., 2022; Caires and Bossolani-Martins, 2023; Cait et al., 2024; Dijkhuizen et al., 2024; Nakhwa et al., 2024; Ratuski et al., 2024). It can also improve sensory function and performance, including whisker touch discrimination (Zheng et al., 2021). In our study, we implemented enrichment intended to increase the use of whisker touch. Since whiskers are critical for guiding forelimb placement in crossing gaps when small mammals climb (Ahl, 1986; Arkley et al., 2014; 2017; Niederschuh et al., 2015; Grant et al., 2018), we reared opossums in cages with periodically varying, spatially complex climbing substrates and intermittently introduced novel toys, since novel objects and other interactive enrichment devices promote active whisking behavior (Sofroniew and Svoboda, 2015; Nakhwa et al., 2024). We show that in a novel setting, both sighted and blind opossums showed differential exploration patterns reflecting the geometry of their rearing environments. Additionally, enriched opossums are faster and more successful in gap crossing using a whisker-dependent strategy. Thus, opossums use behavioral strategies shaped by their rearing environments, and our enrichment approach effectively engages and enhances naturalistic whisker-mediated behavior in both sighted and blind animals, enabling us to evaluate the effects of enriched rearing on compensatory plasticity following early vision loss.

It has been previously reported that early blindness alters the coding of whisker touch inputs in opossums, in a manner consistent with enhanced tactile discrimination, including improved neuronal discriminability and population decoding of whisker stimulus location in S1, as well as lower behavioral texture discrimination thresholds (Ramamurthy and Krubitzer, 2018; Ramamurthy et al., 2021). This involved suppression of whisker-evoked responses combined with increased spatial selectivity, due to the greater relative suppression of neural responses to nonpreferred stimuli (SW) versus the preferred stimulus (BW). These alterations in the receptive fields of neurons in S1 resemble the effects of naturalistic EE on receptive fields of neurons in S1 of adult rats (Polley et al., 1999, 2004). Here we demonstrate that rearing early blind short-tailed opossums in an enriched environment further amplifies these changes. Since selectivity to whisker stimuli was enhanced not just in early blind animals but also in sighted control animals reared in enriched conditions, we can infer that suppression of whisker-evoked responses and increased selectivity of neural responses in the S1 whisker representation are effects of tactile experience rather than visual deprivation itself. Here, we interpret greater selectivity as potentially advantageous for discriminating nearby tactile inputs rather than as a universal improvement in coding, since broader tuning and receptive field overlap can also provide benefits at the population level (Zhang and Sejnowski, 1999; Butts and Goldman, 2006). One general limitation is that recordings were performed under urethane anesthesia, which has been reported in the rodent barrel cortex to produce more pronounced adjacent-whisker responses than in the awake state, such that receptive fields appear broader and laminar differences in receptive field organization are reduced (Simons et al., 1992). However, because the same anesthetic conditions were used for all groups, this is unlikely to affect the comparisons made here.

A key finding was the effect of the rearing environment on the shapes of receptive fields of S1 neurons—shape distributions for both sighted and early blind animals exhibited a prominent shift away from the horizontally anisotropic shapes that dominate in standard-reared opossums. This was accompanied by a reduction in tuning width along row (horizontal) versus arc (vertical) axes in enriched animals—on average, tuning widths were reduced to a greater extent along the row axis, parallel to the main axis of whisker movements. This may reflect more naturalistic patterns of whisker use in short-tailed opossums, enabled by the increase in vertical space and availability of climbing substrates in the enriched rearing environment. In rats, simulations that modeled exploratory behavior predicted changes in the spatial patterns of whisker contact depending on the environment being explored (Hobbs et al., 2015). It was found that whiskers within the same horizontal row were more likely to come in simultaneous contact during exploration of flat surfaces, but in more complex and naturalistic environments, the probability distributions of simultaneous whisker contact shifted away from this row-wise structure. Such alterations in the patterns of tactile stimuli impinging on the whisker array over the course of development may, in turn, impact receptive field structure. In principle, the effects described here could reflect not only altered patterns of passive whisker stimulation associated with greater vertical environmental use but also changes in active vibrissal control and active touch behavior (Mitchinson et al., 2011). These possibilities are not mutually exclusive—rather, greater use of a vertically complex environment may itself alter the timing, vigor, or spatial patterning of whisker movements, thereby changing the tactile signals arriving in S1. Given the close relationship between somatosensory and motor representations in *Monodelphis* (Frost et al., 2000) and broader evidence that S1 plays a role in motor control (Halley et al., 2020), the receptive field changes described here are best interpreted within the broader vibrissal sensorimotor loop (Ahissar and Kleinfeld, 2003) rather than as arising from stimulation pattern alone.

Another possibility is that anisotropic effects on receptive fields are inherent to differences in row versus arc organization within the neocortex. In rats and mice, it is well documented that there is a bias toward preferential connectivity among barrels corresponding to whiskers in the same row (Bernardo et al., 1990; Hoeflinger et al., 1995; Keller and Carlson, 1999; Kim and Ebner, 1999) and neurons in the barrel cortex typically display patterns of activity that tend to be elongated along the row axis (Simons, 1978; Armstrong-James and Fox, 1987; Kleinfeld and Delaney, 1996; Ego-Stengel et al., 2005; Le Cam et al., 2011). Furthermore, there is evidence for differential effects on responses of S1 neurons along row and arc axes when sensory inputs are altered within the whisker system, for example, through chronic stimulation (Quairiaux et al., 2007) or selective trimming (Dubroff et al., 2005; Wallace and Sakmann, 2008) of specific whiskers. Thus, biases in the functional organization and connectivity within the S1 whisker representation may favor plasticity, preferentially sharpening tuning along the row axis.

A long-standing question has been whether reorganization following sensory loss is primarily driven by deprivation of the lost modality or due to increased experience using the spared senses. Studies in blind humans with Braille reading experience support the important role of tactile experience in generating tactile spatial acuity enhancement following blindness (Wong et al., 2011). The relative contributions of deprivation of the lost modality versus increased or modified use of the spared senses (Goldreich and Kanics, 2003; Voss, 2011, 2019; Wong et al., 2011) to different aspects of cortical reorganization following sensory loss are still not well understood. Our study provides clear evidence for the role of experience in driving compensatory neural and behavioral plasticity following sensory loss, highlighting the importance of therapeutic strategies engaging tactile exploration [e.g., through play-based learning of tactile skills (Hilditch, 2024) or the use of haptic devices (Fleck et al., 2025)] to maximize neural and behavioral compensation through touch.

## Synthesis

Reviewing Editor: Arianna Maffei, Stony Brook University Decisions are customarily a result of the Reviewing Editor and the peer reviewers coming together and discussing their recommendations until a consensus is reached. When revisions are invited, a fact-based synthesis statement explaining their decision and outlining what is needed to prepare a revision will be listed below. The following reviewer(s) agreed to reveal their identity: NONE.

The manuscript is considered as well-designed, rigorous and impactful for the field. The reviewers raised several issues that when addressed will improve clarity of the data presentation and add points of discussion of the current findings in the context of previous work.

There are specific questions about the data in Figure 3, including the number of trials that was included and questions about the motivation behind presenting successes and failures.

EB and SC should be defined.

It would also be helpful to test for differences between sEB animals and sSC animals. It appears that sEB animals complete gap crossings more successfully than sSC animals. Assessing this difference may support the conclusion regarding the increased usefulness of whiskers after enucleation.

Clarifications about how responses to different whiskers are measured (in different blocks or interleaved?) would help better understand the experimental design.

Additional information about the strategy for the quantification of receptive fields at the edge of the whisker pad would be helpful.

Details reviewers' comments can be found below.

Reviewer #1

The manuscript describes a study of the impact of environmental complexity, and its interplay with sensory loss, on cortical plasticity. Specifically, a 2x2 study is performed on marsupial Monodelphis domestica comparing early blind and intact animals in conditions of standard and enriched rearing environments. This design allows the researchers to compare and contrast the effects of sensory loss and environmental enrichment in a manner that has not previously been investigated in such a systematic way. The result show adaptive changes with respect to both interventions including behavioural changes and significant alterations to the receptive field properties of S1 whisker-related neurons.

The study is well-designed, carefully executed and analysed and described with suitable detail. I believe it addresses a timely and interesting question and a contributes to the wider debate about the effects of early experience on cortical organisation.

Below is a list of specific queries and comments, that the authors could address:

1. What is the basis for describing greater selectivity as an improvement? It would be helpful if the authors could provide more supportive literature here. The assumption that greater selectivity will lead to better discrimination seems reasonable, but too much selectivity could reduce information as overlap of receptive fields can provide some benefits.

2. it is not possible to discriminate which layer of S1 is being recorded from? Are these likely to be layer IV cells? In rats, layer 4 and 5 cells differ in the extent to which they are sensitive to multiple whiskers (higher in layer 5). I appreciate that the different cortical layers may be less well-defined in M. domestica.

3. Could the reduced response size in s1 neurons of animals raised in enriched environments help explain the greater selectivity, i.e. you might have the same width of bump but less of it is above threshold?

4. Is it possible that changes in anisotrophy could be related to whisker control, rather than stimulation pattern? The authors explain this as possibly resulting from greater vertical movement. It might also be as a result of changes in whisker control which might be visible in the musculature. More generally, there is limited discussion of active vibrissal touch (e.g. Mitchinson et al. 2011) and how that might be impacted by enucleation or enrichment. For instance, enucleation may affect the onset of whisking, affect the development of the whisker musculature, or influence animals to whisk more vigorously. I understand that the manuscript focuses on changes in cortical fields. But wider changes to the vibrissal system would impact on input signals arriving at S1, moreover has S1 likely has direct influences on whisker behavior (Frost et al. 2000) so its role is best considered with the wider loop (see, e.g. Ahissar and Kleinfeld, 2003).

References:

Ahissar, E., &Kleinfeld, D. (2003). Closed-loop neuronal computations: focus on vibrissa somatosensation in rat. Cereb Cortex, 13(1), 53-62.

Frost, S. B., Milliken, G. W., Plautz, E. J., Masterton, R. B., &Nudo, R. J. (2000). Somatosensory and motor representations in cerebral cortex of a primitive mammal (Monodelphis domestica): a window into the early evolution of sensorimotor cortex. J Comp Neurol, 421(1), 29-51.

Mitchinson, B., Grant, R. A., Arkley, K., Rankov, V., Perkon, I., &Prescott, T. J. (2011). Active vibrissal sensing in rodents and marsupials. Philosophical Transactions of the Royal Society B: Biological Sciences, 366(1581), 3037-3048. doi:366/1581/3037 [pii]

Reviewer #2

This paper investigates the impact of early life experience on whisker representations in short-tailed opossums by studying the effects of two manipulations, enriched rearing and binocular enucleation early in life. The study shows that increasing tactile exploration because of environmental complexity and/or loss of vision has measurable behavioral consequences in tasks that are whisker-dependent, and in the whisker representation in S1. This is an interesting study, in particular the effects on the receptive field shape of S1 neurons. The manuscript could be further improved through clarification of a number of issues:

- Both the small and large gaps are specified as ranges. Are different gap sizes tested with different animals, or does every animal experience a range? Why is the division between the 2 sizes placed at 10cm?

- It seems that sEB animals complete gap crossings more successfully than sSC animals? It would be useful to test for differences between these two groups since it would support the conclusion regarding the increased usefulness of whiskers after enucleation.

- Regarding Figure 3B, it is a bit confusing to show both failure and success rates since they have to sum to 100%. It might be better to reduce this figure to just the success rate.

- How many trials were collected per animal for the data in Fig. 3? The distributions are very oddly shaped for some of the groups, and suggest that only limited data were available? In general, it would be very useful to increase the sample size beyond 3-4 animals per group for all of the behavioral tests to increase the power of the statistical tests.

- Are responses to different whiskers measured in different blocks or randomly interleaved? If they are measured in different blocks, it would be useful to at least comment on stationarity of anesthesia throughout.

- In general, it would be useful to at least discuss possible consequences of using urethane as anesthetic agent. Since it was used for all groups, it should not impact any of the comparisons, but it would still be useful to at least discuss that previous studies have shown that receptive fields appear larger under urethane anesthesia.

- How are receptive fields quantified for whiskers that are located at the edge of the whisker pad and therefore have less surround whiskers?

- Is it possible that the lower overall firing rate for animals raised in enriched environments made the responses of surrounding whiskers harder to detect?

- SC and EB are not defined, as far as I can tell.
