# Supplementary material for: Complex Three-Dimensional Rearing Environments Amplify Compensatory Plasticity Following Early Blindness
Source: eNeuro. 2026 Jul 21;13(7):ENEURO.0059-26.2026. doi: 10.1523/ENEURO.0059-26.2026 (PMC13406312; doi:10.1523/ENEURO.0059-26.2026)
Supplement: Table 2-1 — ANOVA marginal tests for fixed effects in Table 2. Download Table 2-1, DOCX file. [file eneuro-13-ENEURO.0059-26.2026-s011.docx]

**Extended Data Table 2-1. ANOVA marginal tests for fixed effects in Table 2.**

| **Figure 3C** | **ANOVA marginal tests: DFMethod = 'residual'**  Term FStat DF1 DF2 pValue  {'(Intercept)'} 23.444 1 26 5.1009e-05 {'ExptGp' } 0.7076 3 26 0.55618 {'Gap' } 84.94 1 26 1.1275e-09 {'Trim' } 0.39839 1 26 0.53343 {'ExptGp:Gap' } 1.6607 3 26 0.19987 {'ExptGp:Trim'} 2.5971 3 26 0.073804 |
| --- | --- |
| **Figure 3D** | **ANOVA marginal tests: DFMethod = 'residual'** Term FStat DF1 DF2 pValue  {'(Intercept)'} 265.73 1 10508 5.1219e-59 {'ExptGp' } 227.57 1 10508 6.8796e-51 {'Gap' } 363.44 1 10508 1.1011e-79 {'Trim' } 26.357 1 10508 2.888e-07 {'ExptGp:Gap' } 268.66 1 10508 1.2153e-59 {'ExptGp:Trim'} 6.2261 1 10508 0.012603 |

F statistics, numerator and denominator degrees of freedom (DF1, DF2), and p-values are reported for each model term in analyses corresponding to **Table 2** and **Figure 3**.
