# Supplementary material for: Complex Three-Dimensional Rearing Environments Amplify Compensatory Plasticity Following Early Blindness
Source: eNeuro. 2026 Jul 21;13(7):ENEURO.0059-26.2026. doi: 10.1523/ENEURO.0059-26.2026 (PMC13406312; doi:10.1523/ENEURO.0059-26.2026)
Supplement: Table 3-1 — ANOVA marginal tests for fixed effects in Table 3. Download Table 3-1, DOCX file. [file eneuro-13-ENEURO.0059-26.2026-s012.docx]

**Extended Data Table 3-1. ANOVA marginal tests for fixed effects in Table 3.**

| **Figure 4C** | **ANOVA marginal tests: DFMethod = 'Satterthwaite'**  Term FStat DF1 DF2 pValue  {'(Intercept)'} 39.173 1 339 1.1718e-09 {'ExptGp' } 0.25773 3 339 0.85581 |
| --- | --- |
| **Figure 4D** | **ANOVA marginal tests: DFMethod = 'Satterthwaite'**  Term FStat DF1 DF2 pValue  {'(Intercept)'} 140.79 1 4.7301 0.00010652 {'ExptGp' } 17.101 3 232.42 4.5371e-10 |
| **Figure 4E** | **ANOVA marginal tests: DFMethod = 'Satterthwaite'**  Term FStat DF1 DF2 pValue  {'(Intercept)'} 266.75 1 233 1.7587e-40 {'ExptGp' } 12.369 3 233 1.5486e-07 |
| **Figure 4F** | **ANOVA marginal tests: DFMethod = 'Satterthwaite'**  Term FStat DF1 DF2 pValue  {'(Intercept)'} 1188.9 1 3483 2.0524e-224 {'ExptGp' } 40.206 3 3483 1.5495e-25 |

F statistics, numerator and denominator degrees of freedom (DF1, DF2), and p-values are reported for each model term in analyses corresponding to **Table 3** and **Figure 4**.
