# Supplementary material for: Complex Three-Dimensional Rearing Environments Amplify Compensatory Plasticity Following Early Blindness
Source: eNeuro. 2026 Jul 21;13(7):ENEURO.0059-26.2026. doi: 10.1523/ENEURO.0059-26.2026 (PMC13406312; doi:10.1523/ENEURO.0059-26.2026)
Supplement: Table 3-2 — ANOVA marginal tests for fixed effects in Figure 5. Download Table 3-2, DOCX file. [file eneuro-13-ENEURO.0059-26.2026-s013.docx]

**Extended Data Table 3-2. ANOVA marginal tests for fixed effects in Figure 5.**

| **Figure 5C** | **ANOVA marginal tests: DFMethod = 'Satterthwaite'**  Term FStat DF1 DF2 pValue  {'(Intercept)'} 8.7766 1 169 0.0034913 {'ExptGp' } 4.6638 3 169 0.0037027 |
| --- | --- |
| **Figure 5D** | **ANOVA marginal tests: DFMethod = 'Satterthwaite'**  Term FStat DF1 DF2 pValue  {'(Intercept)'} 27.068 1 233 4.3037e-07 {'ExptGp' } 8.1681 3 233 3.4152e-05 |
| **Figure 5E (left)** | **ANOVA marginal tests: DFMethod = 'Satterthwaite'**  Term FStat DF1 DF2 pValue  {'(Intercept)'} 146.11 1 233 1.9597e-26 {'ExptGp' } 2.7797 3 233 0.041847 |
| **Figure 5E (right)** | **ANOVA marginal tests: DFMethod = 'Satterthwaite'**  Term FStat DF1 DF2 pValue  {'(Intercept)'} 391.43 1 233 8.7398e-52 {'ExptGp' } 37.602 3 233 7.4223e-20 |
| **Figure 5F (left)** | **ANOVA marginal tests: DFMethod = 'Satterthwaite'**  Term FStat DF1 DF2 pValue  {'(Intercept)'} 156.86 1 233 7.4003e-28 {'ExptGp' } 34.262 3 233 2.2108e-18 |
| **Figure 5F (right)** | **ANOVA marginal tests: DFMethod = 'Satterthwaite'**  Term FStat DF1 DF2 pValue  {'(Intercept)'} 461.91 1 233 3.2951e-57 {'ExptGp' } 2.7633 3 233 0.042756 |

F statistics, numerator and denominator degrees of freedom (DF1, DF2), and p-values are reported for each model term in analyses corresponding to **Table 3** and **Figure 5**.
