# Supplementary figures and images for: Complex Three-Dimensional Rearing Environments Amplify Compensatory Plasticity Following Early Blindness
Source: eNeuro. 2026 Jul 21;13(7):ENEURO.0059-26.2026. doi: 10.1523/ENEURO.0059-26.2026 (PMC13406312; doi:10.1523/ENEURO.0059-26.2026)

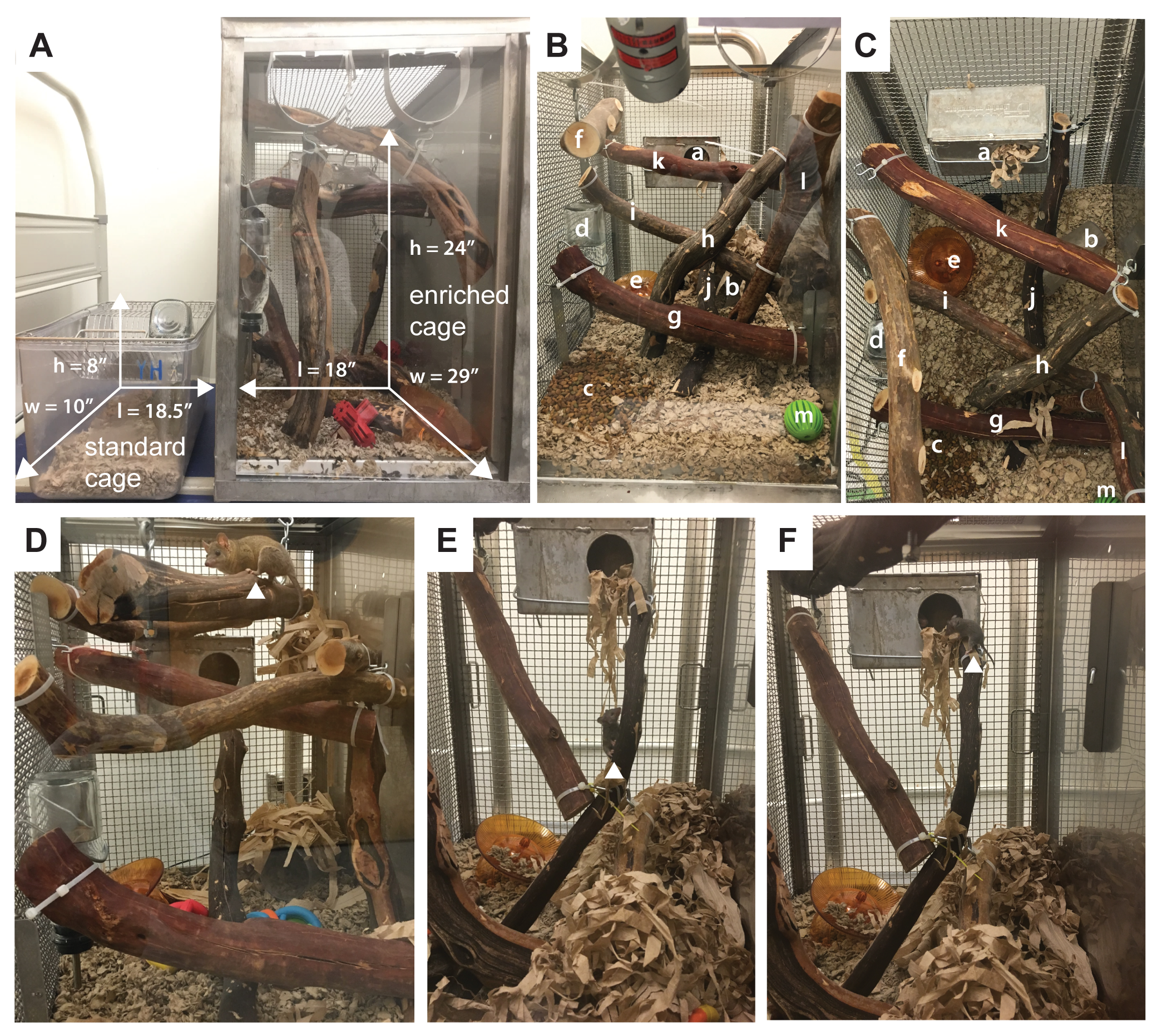

Supplement: Figure 1-1 — Standard and enriched rearing environments used for short-tailed opossums. A. Comparison of cages used in standard (left) and enriched (right) rearing paradigms. Enriched cages (29”l x 18”w x 24”h) were substantially larger (∼8x in volume) than standard cages (8.5”l x 10”w x 8”h), especially in the vertical dimension (∼3x). Each enriched cage contained a nesting box positioned high above the ground, in addition to the standard nesting cup on the cage floor. Cages also included various enrichment objects: regularly repositioned manzanita branches, rotating sets of enrichment toys, a running wheel, and social housing (see Materials and Methods). B. Frontal view of an enriched cage. C. Top view of the same enriched cage shown in (B). Letters mark individual components visible in both views: nesting box (a), nesting cup (b), food (c), water (d), running wheel (e), manzanita branches (f–l), and enrichment toys (m). D. Mother with an attached experimental litter after placement in the enriched rearing environment. E–F. Early blind weanling climbing up a branch to reach the nesting box. Download Figure 1-1, TIF file. [file eneuro-13-ENEURO.0059-26.2026-s007.tif]

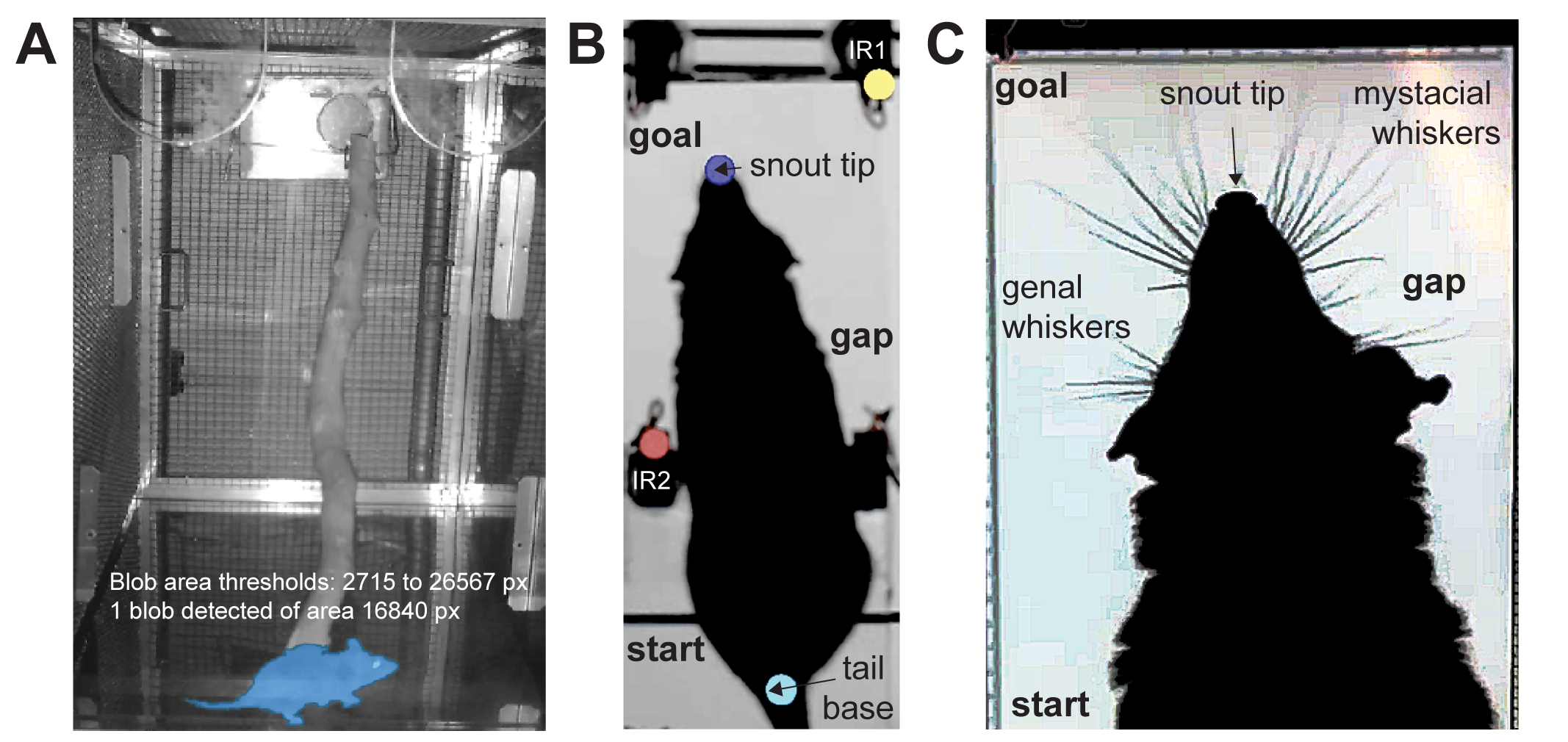

Supplement: Figure 2-1 — Behavioral movie analyses. A. Representative frame showing semi-automated identification of an opossum during arena testing using idtracker.ai. B. Representative frame showing behavioral tracking in the gap crossing task using DeepLabCut. “IR1” and “IR2” indicate the positions of infrared motion sensors mounted on the edge of each platform, tracked with DeepLabCut to aid in gap crossing analyses. C. Zoomed-in view showing an opossum on the start platform extending its whiskers toward the goal platform prior to crossing the gap. Download Figure 2-1, TIF file. [file eneuro-13-ENEURO.0059-26.2026-s008.tif]

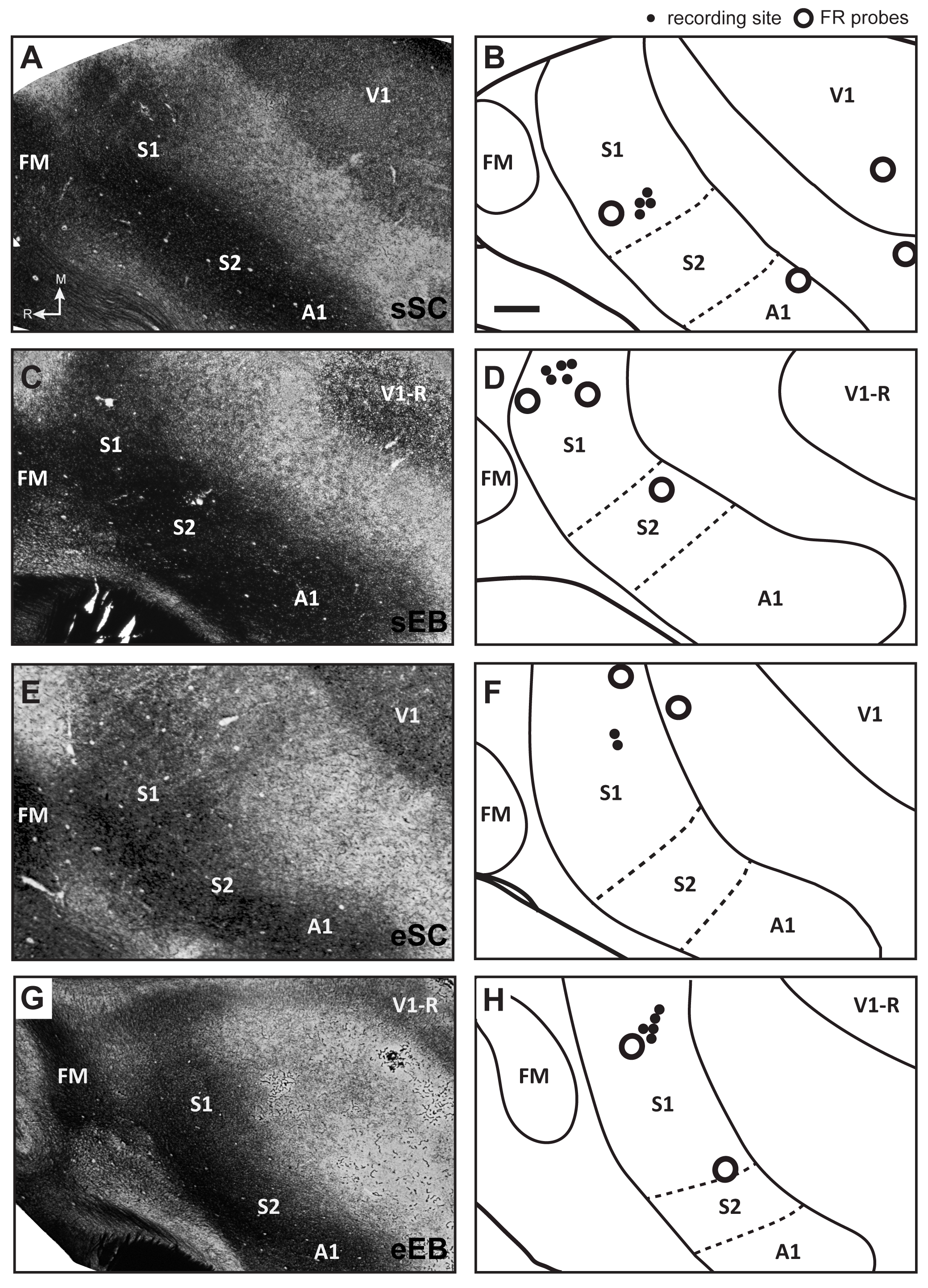

Supplement: Figure 4-1 — Histological verification of recording sites. A, C, E, G. Myelin-stained tangential sections of cortex from a standard-reared sighted (sSC) animal (A), a standard-reared early blind (sEB) animal (C), an enriched sighted (eSC) animal (E), and an enriched early blind (eEB) animal (G). Primary somatosensory cortex (S1) is identifiable as a darkly staining region relative to adjacent cortical fields. B, D, F, H. Reconstructions of myeloarchitectural borders drawn from the series of myelin-stained sections in sSC (B), sEB (D), eSC (F) and eEB (H) animals. The locations of fluorescent probe insertions are shown as open circles, and recording sites are shown as black filled circles. Only sites at which whisker-evoked neuronal responses were quantified are included. In all panels, medial (M) and rostral (R) directions are indicated by arrows. Scale bar = 250 µm. Download Figure 4-1, TIF file. [file eneuro-13-ENEURO.0059-26.2026-s010.tif]
